# The Inflammaging-Redox-InflammamiR Axis in Metabolic Aging: From Diagnostic Clusters to Integrated Risk Phenotypes

**DOI:** 10.3390/biom16071008

**Published:** 2026-07-10

**Authors:** Nurzhanyat Ablaikhanova, Ingkar Okhas, Aidos Bolatov, Beibarys Mukhitdin, Zhazira Zhunusbayeva, Gulmira Assan, Marzhan Kulbayeva, Anar Tolebaeva, Arailym Yessenbekova, Iryna Rusanova

**Affiliations:** 1 Department of Biophysics, Biomedicine and Neuroscience, Farabi University, 050000 Almaty, Kazakhstan; nurzhanat.ablaihanova@kaznu.kz (N.A.); okhas_ingkar2@live.kaznu.kz (I.O.); gulmiraasan28@gmail.com (G.A.); marzhan.kulbaeva@kaznu.edu.kz (M.K.); tleuberdiasha@gmail.com (A.T.); 2 Department of Science, “University Medical Center” Corporate Fund, 010000 Astana, Kazakhstan; bolatovaidos@gmail.com; 3 Shenzhen University Medical School, Shenzhen University, Shenzhen 518060, China; mukhitdin00beibarys@gmail.com; 4 Laboratory of Environmental Physiology of Humans and Animals, Institute of Genetics and Physiology, 050000 Almaty, Kazakhstan; 5 Department of Molecular Biology and Genetics, Farabi University, 050000 Almaty, Kazakhstan; zhazira.zhunusbayeva@kaznu.kz; 6 Department of Normal Physiology with the Course of Biophysics, Asfendiyarov Kazakh National Medical University, 050000 Almaty, Kazakhstan; 7 Department of Biochemistry and Molecular Biology I, Faculty of Science, University of Granada, 18019 Granada, Spain; 8 Centro de Investigación Biomédica en Red de Fragilidad y Envejecimiento Saludable (CIBERFES), Instituto de Salud Carlos III (ISCIII), 28029 Madrid, Spain; 9 Centro de Investigación Biomédica, ParqueTecnológico de Ciencias de la Salud, Universidad de Granada, 18016 Granada, Spain; 10 Instituto de Investigación Biosanitaria (IBS Granada), Hospital Unversitario San Cecilio, 18016 Granada, Spain

**Keywords:** inflammaging, oxidative stress, inflammamiRs, metabolic syndrome, aging, microRNAs, biomarkers

## Abstract

Age-associated metabolic dysfunction is commonly defined by abnormalities in adiposity, glucose regulation, lipid metabolism, and blood pressure. Although clinically useful, these criteria do not fully capture the biological heterogeneity that explains why older adults with similar metabolic profiles may follow divergent trajectories toward type 2 diabetes, cardiovascular disease, metabolic dysfunction-associated steatotic liver disease, frailty or multimorbidity. This narrative Review summarizes clinical, translational, and mechanistic evidence on the biological processes that shape metabolic aging, with particular emphasis on inflammaging, immunosenescence, cellular senescence, oxidative stress, mitochondrial dysfunction, adipose tissue dysfunction, endothelial injury, and inflammation-related microRNAs. We first discuss how chronic low-grade inflammation and immune remodeling alter the interpretation of conventional metabolic syndrome components in older adults. We then review redox imbalance and mitochondrial stress as amplifiers of insulin resistance, lipid injury, vascular dysfunction, and tissue remodeling. The review also examines inflammation-related microRNAs, including circulating and extracellular-vesicle-associated miRNAs, as post-transcriptional regulators that may connect inflammatory, metabolic, and redox pathways. Finally, we discuss how conventional metabolic markers may be integrated with inflammatory mediators, oxidative-stress indicators, adipokines, endothelial and senescence-related markers, and miRNA profiles to improve biological interpretation of metabolic risk. Within this context, we present the Inflammaging–Redox–InflammamiR Axis as a conceptual framework for organizing these overlapping mechanisms rather than as an established diagnostic or causal model. The proposed biomarker tiers and candidate risk phenotypes are author-derived, hypothesis-generating constructs intended to guide future longitudinal and interventional research. Clinical translation will require standardized assays, longitudinal validation, external replication, and intervention studies.

## 1. Introduction

Metabolic syndrome (MetS) is conventionally defined as a clustering of cardiometabolic risk factors, including central adiposity, elevated blood pressure, dysglycemia, hypertriglyceridemia and reduced high-density lipoprotein cholesterol content (HDL-C), with insulin resistance often considered a central pathophysiological feature rather than a required diagnostic criterion in all definitions [1,2]. This clinical construct remains valuable because it identifies individuals at increased risk of type 2 diabetes mellitus (T2DM), cardiovascular disease, chronic kidney disease, and premature mortality [1]. Yet, in older adults, metabolic dysfunction rarely represents an isolated disturbance of glucose, lipid, or blood-pressure regulation. Instead, it emerges within a broader biological landscape shaped by immune remodeling, cellular senescence, and chronic low-grade inflammation [3,4], together with mitochondrial impairment, oxidative stress [5], vascular aging and altered adipose tissue biology [6].

This age-dependent context helps explain why apparently similar metabolic profiles may lead to divergent clinical trajectories. Inflammaging, broadly defined as a chronic, systemic, and low-grade inflammatory state that develops with aging, is associated with increased circulating inflammatory mediators and greater vulnerability to age-related disease [3]. Cellular senescence contributes through the senescence-associated secretory phenotype (SASP), which can amplify local and systemic inflammation [4]. In parallel, immunosenescence reduces immune adaptability while preserving or intensifying basal inflammatory tone, creating a biological environment in which metabolic stress is more likely to progress toward tissue injury, frailty, and multimorbidity [7].

Adipose tissue is a key interface between aging and metabolic disease. Beyond energy storage, adipose tissue functions as an endocrine and immune-active organ that regulates systemic metabolism through adipokines, cytokines, lipid flux, and immune-cell interactions [8]. With aging, adipose tissue undergoes impaired adipogenesis, redistribution, immune-cell infiltration, fibrosis, mitochondrial stress, and altered adipokine secretion, reducing its capacity to store excess lipids safely and promoting ectopic lipid deposition, insulin resistance and endothelial dysfunction [9,10]. Thus, central obesity in older adults may indicate not only excess adiposity, but also adipose tissue dysfunction, inflammatory activation and reduced metabolic flexibility [6,11].

Mitochondrial dysfunction and oxidative stress provide another mechanistic bridge between aging and cardiometabolic injury. Mitochondria are central hubs for energy production, redox signaling and inflammatory regulation; when mitochondrial function declines, increased reactive oxygen species generation, impaired bioenergetics and altered stress signaling can reinforce inflammation and metabolic dysfunction [5]. Oxidative stress contributes to insulin resistance, lipid peroxidation, protein oxidation, endothelial dysfunction, and vascular injury [12]. These mechanisms suggest that age-associated metabolic dysfunction is not merely a downstream consequence of excess adiposity or hyperglycemia, but a systems-level disorder involving reciprocal interactions between metabolism, inflammation, mitochondrial function, and redox balance [5,13].

Conventional cardiometabolic risk assessment relies on fasting glucose, glycated hemoglobin (HbA1c), insulin or insulin-resistance indices, triglycerides, HDL-C, low-density lipoprotein cholesterol content (LDL-C), blood pressure, waist circumference, body mass index, and related anthropometric indicators [14]. These markers are clinically indispensable, but they mainly capture established metabolic phenotypes rather than the upstream biological processes that shape vulnerability, resilience, and progression [15]. This limitation is particularly important in aging populations, where chronological age does not adequately reflect biological age or metabolic resilience [7].

A more informative approach may require integrating conventional metabolic markers with inflammatory, oxidative-stress, mitochondrial, and post-transcriptional regulatory biomarkers. Circulating inflammatory mediators such as C-reactive protein (CRP), interleukin-6 (IL-6), tumor necrosis factor-α (TNF-α), and interleukin-1β (IL-1β) have been widely studied in aging, metabolic disease, and frailty [7,16]. Oxidative-stress biomarkers, including indices of lipid peroxidation, protein oxidation, and antioxidant defense, may provide complementary information on redox injury and endothelial-metabolic damage [12]. Mitochondrial stress-related markers such as growth differentiation factor 15 (*GDF15*) have also attracted interest in aging biology, although their interpretation remains complex because they may reflect both tissue injury and adaptive stress responses [17].

Inflammation-related microRNAs (miRNAs), often termed inflammamiRs, add a further regulatory layer. miRNAs are small non-coding RNAs that regulate gene expression post-transcriptionally, and several miRNAs are involved in inflammatory, metabolic, and stress-response pathways [18]. miR-21-5p, miR-146a-5p and miR-155 are among the most frequently discussed inflammamiRs in aging and metabolic disease because they intersect with nuclear factor-κB (NF-κB), inflammasome signaling, insulin sensitivity, adipose inflammation, and immune-cell activation [19,20,21,22]. However, their biological meaning depends on tissue source, disease stage, inflammatory burden, and interaction with other metabolic and redox pathways [19,23].

This review proposes the Inflammaging–Redox–InflammamiR Axis as a conceptual framework to integrate inflammation, oxidative stress, and miRNAs in metabolic aging. Rather than a validated clinical model, it is a hypothesis-generating approach that suggests these interacting biomarkers may improve risk stratification beyond traditional metabolic criteria, but it still requires further validation.

### Literature Search Strategy and Evidence Synthesis

This narrative review was designed as a conceptual synthesis rather than as a systematic review or meta-analysis. The bibliographic search was performed using major electronic databases, including PubMed/MEDLINE, Scopus, and Web of Science, covering publications from January 2010 to March 2026, with particular emphasis on high-impact studies published between 2023 and 2026. Search terms were combined using topic-specific clusters related to metabolic dysfunction, aging biology, inflammation, redox stress, and non-coding RNA regulation. These included “metabolic syndrome,” “meta-bolic aging,” “insulin resistance,” “type 2 diabetes,” “cardiometabolic disease,” “MASLD,” “inflammaging,” “immunosenescence,” “cellular senescence,” “senes-cence-associated secretory phenotype,” “oxidative stress,” “redox biology,” “mitochon-drial dysfunction,” “NLRP3 inflammasome,” “microRNA,” “miRNA,” “inflammamiR,” “extracellular vesicles,” “biomarkers,” “frailty,” “multimorbidity,” “biological aging,” “multi-omics,” and “machine learning.” Boolean operators (AND/OR) were used to refine the search strategy. Additional articles were identified by screening the reference lists of key reviews and highly relevant primary studies.

We prioritized peer-reviewed systematic reviews, meta-analyses, narrative reviews, human cohort studies, translational biomarker studies, mechanistic experimental studies, and multi-omics analyses that addressed the relationship between aging, metabolic dysfunction, inflammation, oxidative stress, mitochondrial biology, miRNA regulation or biomarker-based risk stratification. Studies with limited relevance, duplicated findings, or insufficient methodological quality were excluded. The final selection prioritized studies with strong mechanistic rationale, translational relevance, and potential contribution to the conceptual development of the proposed Inflammaging–Redox–InflammamiR Axis.

## 2. Inflammaging as the Immune Substrate of Metabolic Aging

### 2.1. Definition and Biological Basis of Inflammaging

Inflammaging describes the chronic, systemic, and low-grade inflammatory state that develops with advancing age in the absence of overt infection, acute tissue injury, or clinically evident inflammatory disease [3]. It is now regarded as a central mechanism of geroscience because it links biological aging with several age-associated conditions, including T2DM mellitus, cardiovascular disease, chronic kidney disease, frailty, sarcopenia, and multimorbidity [24]. Unlike acute inflammation, which is usually transient and directed toward host defense or tissue repair, inflammaging is persistent, subclinical, and self-reinforcing. It arises from the cumulative interaction of immune remodeling, unresolved cellular stress, metabolic overload, mitochondrial dysfunction, cellular senescence, and impaired inflammatory resolution [3,4].

A major biological contributor to inflammaging is immunosenescence, which refers to age-related remodeling of innate and adaptive immunity rather than simple immune decline. Aging is associated with reduced naïve T-cell output, restricted immune-receptor diversity, impaired vaccine responses, altered macrophage and neutrophil function, and expansion of differentiated or exhausted immune-cell populations [25,26]. This produces a paradoxical immune phenotype: adaptive immune precision declines, whereas basal innate immune activation and inflammatory signaling increase [25]. Consequently, older adults may be simultaneously more vulnerable to infection and less able to terminate chronic inflammatory responses [24,27].

Cellular senescence is another key source of age-related inflammation. Senescent cells are metabolically active cells that have permanently exited the cell cycle in response to replicative exhaustion, DNA damage, oxidative stress, mitochondrial dysfunction, or other stress signals [4]. Although senescence has physiological roles in tumor suppression and wound healing, age-related accumulation of senescent cells becomes harmful when their immune clearance is inefficient [28]. These cells acquire a senescence-associated secretory phenotype (SASP), characterized by increased secretion of cytokines, chemokines, growth factors, matrix-remodeling enzymes, and proteases, including IL-6, IL-1β, and TNF-α [4]. Through these SASP-associated secretory factors, senescent cells amplify tissue inflammation, induce secondary senescence, and disturb extracellular-matrix and metabolic homeostasis [28].

At the molecular level, inflammaging is sustained by interconnected inflammatory pathways. NF-κB integrates signals from cytokines, oxidative stress, metabolic stress, pattern-recognition receptors, and senescence-associated damage signals. The nucleotide-binding oligomerization domain-like receptor family pyrin domain-containing 3 (NLRP3) inflammasome promotes caspase-1 activation and maturation of IL-1β and IL-18, thereby linking sterile inflammation with insulin resistance, β-cell stress, and age-related metabolic disease [3,4]. These mechanisms explain why CRP, IL-6, TNF-α, IL-1β, IL-18, and chemokines are repeatedly associated with aging, frailty, and cardiometabolic risk [16].

Adipose tissue provides a particularly important bridge between inflammaging and metabolic dysfunction. In older adults, adipose tissue undergoes impaired adipogenesis, adipocyte hypertrophy, fibrosis, mitochondrial stress, altered adipokine secretion, macrophage infiltration, and accumulation of senescent cells [29,30]. These changes promote chronic adipose inflammation, impair lipid buffering and contribute to systemic insulin resistance. Thus, central adiposity in later life should be interpreted not only as excess fat mass, but also as a potential marker of adipose immune activation and endocrine dysfunction [10].

Together, these processes create a self-perpetuating inflammatory loop in which metabolic stress, oxidative damage and senescent-cell accumulation generate inflammatory signals, while persistent inflammation further worsens insulin resistance, vascular dysfunction, and tissue injury [3,4]. Inflammaging therefore provides the biological background in which metabolic dysfunction in older adults becomes more heterogeneous, inflammatory, and clinically unstable than in younger populations. This explains why conventional metabolic markers may not fully capture risk and why inflammatory biomarkers can provide biologically meaningful information for distinguishing stable metabolic aging from high-risk inflammatory metabolic dysfunction.

### 2.2. Major Inflammatory Biomarkers

Inflammatory biomarkers in metabolic aging are most informative when they are interpreted as pathway-level signals rather than as isolated diagnostic tests. In older adults, the same clinical phenotype—central adiposity, dysglycemia, hypertriglyceridemia, low HDL-C or elevated blood pressure—may arise from different inflammatory architectures. Some individuals show predominantly systemic acute-phase activation, whereas others have adipose tissue inflammation, inflammasome-driven cytokine production, immune-cell recruitment, endothelial activation, or inflammatory-fibrotic remodeling. The biomarkers summarized in Table 1 therefore represent complementary layers of the inflammaging-metabolic dysfunction axis rather than interchangeable measures of “inflammation”.

CRP, particularly high-sensitivity CRP (hs-CRP), remains the most clinically tractable marker of low-grade systemic inflammation. Its value lies less in biological specificity than in analytical robustness: hs-CRP is inexpensive, standardized, and widely available. In metabolic aging, elevated hs-CRP suggests that adiposity, insulin resistance, or vascular risk has become embedded within a broader inflammatory state. However, as a downstream acute-phase protein, CRP cannot localize inflammation to adipose tissue, vasculature, liver, or occult comorbidity, and is readily influenced by infection, smoking, and autoimmune disease. Thus, hs-CRP is useful as a first-line inflammatory risk signal, but insufficient as a stand-alone mechanistic biomarker [31,32].

IL-6 provides a more proximal signal of inflammatory aging by linking immune-cell activation, senescent-cell secretory activity, adipose inflammation and hepatic CRP synthesis. Comparative analyses in older adults suggest that IL-6 may outperform TNF-α and IL-1β (IL-1β) as a peripheral marker of chronic inflammation in multimorbid aging [16]. Yet IL-6 is pleiotropic: sustained elevation may indicate inflammatory metabolic stress, whereas transient skeletal-muscle-derived IL-6 during exercise may be adaptive. IL-6 therefore requires interpretation alongside CRP, adiposity, metabolic profile, and clinical context.

TNF-α and IL-1β capture more pathway-specific inflammatory biology. TNF-α is linked to adipose macrophage activation, NF-κB and c-Jun N-terminal kinase (JNK) signaling, impaired insulin receptor signaling and obesity-related insulin resistance. Clinical evidence also associates TNF-α with metabolic disturbance and aging-related phenotypes [43]. IL-1β, generated after inflammasome activation, is closely connected to β-cell stress, impaired insulin secretion, and sterile inflammation, consistent with therapeutic interest in IL-1 signaling in obesity and T2DM [44]. However, circulating TNF-α and IL-1β are often low, variable, and weak proxies for local tissue inflammation.

The NLRP3 inflammasome provides an upstream framework for IL-1β and IL-18. By sensing lipotoxicity, cholesterol crystals, mitochondrial reactive oxygen species, and cellular stress, NLRP3 promotes caspase-1-dependent maturation of IL-1β and IL-18. Evidence continues to place NLRP3 at the intersection of obesity-induced inflammation, insulin resistance, and T2DM-related vascular complications [45,46]. IL-18 is more stable than IL-1β and has been associated with MetS, T2DM risk, and cardiometabolic disease, but both IL-18 and inferred NLRP3 activity are influenced by infection, autoimmunity, renal function, and liver disease [47].

Chemokines reflect inflammatory cell trafficking rather than cytokine burden alone. Monocyte chemoattractant protein-1/C-C motif chemokine ligand 2 (MCP-1/CCL2) recruits *CCR2*-positive monocytes into inflamed or metabolically stressed tissues [48,49]. In obesity and metabolic aging, adipocyte hypertrophy promotes chemokine production, macrophage accumulation, cytokine release, and impaired insulin sensitivity in visceral adipose tissue [50,51]. Clinical data further link MCP-1/CCL2 biology with obesity in T2DM [52], although circulating MCP-1/CCL2 is not adipose-specific and also reflects vascular, renal and neuroinflammatory processes [49].

Adipokines connect inflammatory signaling with adipose tissue function. Adiponectin reflects metabolically healthier adipose tissue and exerts insulin-sensitizing, anti-inflammatory and lipid-regulatory effects, whereas low adiponectin is associated with visceral adiposity, insulin resistance, MetS, T2DM and metabolic dysfunction-associated steatotic liver disease (MASLD) [53,54]. Leptin reflects fat mass and hypothalamic–immune signaling, but hyperleptinemia in obesity often indicates leptin resistance [39]. Accordingly, the leptin/adiponectin ratio may better capture adipose endocrine imbalance than either marker alone [39,55,56]. Resistin, PAI-1, encoded by *SERPINE1*, retinol-binding protein 4, visfatin and chemerin may further refine this axis, although clinical translation is constrained by heterogeneity, tissue-source ambiguity, and assay variability [57,58,59].

Finally, galectin-3 captures inflammatory–fibrotic remodeling relevant to advanced metabolic aging. Population-based and metabolomic studies link galectin-3 pathways with diabetes, MetS, triglyceride metabolism, insulin-related traits, and incident cardiometabolic disease [60,61,62]. Its value may be greatest when metabolic dysfunction overlaps with heart failure, chronic kidney disease, or vascular remodeling, but this breadth also limits specificity because galectin-3 is influenced by cardio-renal disease, fibrosis, malignancy, and systemic inflammation [63].

Overall, these biomarkers are best viewed as partially overlapping pathway signals: hs-CRP and IL-6 reflect systemic inflammatory burden; TNF-α, IL-1β, NLRP3, and IL-18 indicate cytokine–inflammasome activity; MCP-1/CCL2 captures immune-cell recruitment; adipokines describe adipose endocrine–inflammatory dysfunction; and galectin-3 reflects inflammatory–fibrotic remodeling. Their value in metabolic aging is therefore likely to emerge from integrated biomarker patterns rather than single thresholds.

### 2.3. Sources of Inflammation in Older Adults

Inflammation in older adults is not generated by a single tissue or pathway. Rather, it reflects the cumulative output of metabolically stressed organs, immune-remodeled tissues, and comorbidity-related inflammatory signals. Visceral adipose tissue is one of the most important contributors because it combines lipid-buffering failure with immune activation. In insulin-resistant states, human visceral adipose tissue shows impaired vascular-remodeling gene programs, suggesting that limited adaptive expansion and tissue perfusion may precede or accompany inflammatory dysfunction [64]. In parallel, adipocyte hypertrophy, hypoxia, fibrosis, and immune-cell infiltration promote cytokine and chemokine production, thereby converting visceral adipose tissue from a lipid-storage depot into an active inflammatory organ [65,66]. This process is particularly relevant in aging, when adipose tissue plasticity declines and ectopic lipid deposition becomes more likely.

Senescent and dysfunctional immune cells add a second layer of inflammatory pressure. Aging reshapes both innate and adaptive immunity, but its metabolic consequences are especially evident when immune cells accumulate in adipose tissue, liver, skeletal muscle, and vascular compartments. Recent experimental evidence indicates that age-related changes in the visceral adipose immune microenvironment can directly influence insulin sensitivity, showing that immune composition within adipose tissue is not merely a correlate of aging but can shape metabolic function [67]. This reinforces the idea that metabolic aging depends not only on the quantity of adipose tissue, but also on the inflammatory quality of its immune niche.

The vascular endothelium is another major source and amplifier of inflammatory metabolic risk. Endothelial cells exposed to hyperglycemia, excess fatty acids, oxidized lipids, and disturbed hemodynamics acquire a pro-inflammatory and pro-adhesive phenotype, characterized by impaired nitric oxide signaling, increased leukocyte recruitment and reduced vascular repair capacity. In metabolic aging, endothelial inflammation is clinically important because it links systemic metabolic stress to arterial stiffness, microvascular rarefaction, hypertension, and atherosclerotic disease [68,69,70]. Thus, the endothelium should be viewed not only as a target of inflammation, but also as an active participant in propagating cardiometabolic injury.

Skeletal muscle also contributes to inflammatory metabolic aging. As the major site of insulin-stimulated glucose disposal, skeletal muscle strongly determines whole-body insulin sensitivity. Aging-related loss of muscle mass and quality, intramuscular lipid accumulation, mitochondrial dysfunction and reduced contractile activity impair glucose uptake and metabolic flexibility. Inflammation can further compromise muscle insulin signaling, while physical inactivity and sarcopenia reduce the release of metabolically beneficial myokines [71,72]. Conversely, myokines released during muscle contraction can improve insulin sensitivity and regulate glucose–lipid metabolism, highlighting skeletal muscle as both a target and regulator of systemic inflammation [73].

Liver-adipose tissue crosstalk provides a further inflammatory axis. In visceral obesity, increased fatty acid flux from adipose tissue to the liver promotes hepatic lipid accumulation, mitochondrial stress, endoplasmic reticulum stress, and inflammatory signaling. The liver, in turn, releases hepatokines, inflammatory mediators and atherogenic lipid particles that worsen insulin resistance in adipose tissue, skeletal muscle and vasculature. This inter-organ loop is central to MASLD, which is increasingly understood as a systemic metabolic disorder rather than an isolated hepatic condition [74,75]. Recent work on MASLD emphasizes that metabolic fluxes across the gut–adipose tissue–liver axis shape hepatic inflammation, insulin resistance and disease progression [76].

The gut microbiome represents another source of inflammatory metabolic stress. Aging, diet, reduced physical activity, medications, and multimorbidity can alter microbial composition and weaken intestinal barrier function. This may facilitate low-grade translocation of lipopolysaccharide and other microbial products, a process often referred to as metabolic endotoxemia. These microbial products activate innate immune pathways, including TLR4 and NF-κB signaling, thereby promoting systemic inflammation, insulin resistance and dyslipidemia [77,78]. Lifespan-focused reviews further suggest that age-related microbiome changes interact with obesity and metabolic disease, making the gut an important contributor to inflammatory heterogeneity in older adults [79].

Finally, comorbidities and polypharmacy modify the inflammatory background against which metabolic dysfunction develops. Chronic kidney disease, heart failure, osteoarthritis, chronic liver disease, sleep disorders, and depression can each raise inflammatory tone, while multimorbidity increases the probability that circulating biomarkers reflect several overlapping disease processes rather than one metabolic pathway [80,81,82]. Polypharmacy may further influence inflammatory metabolism through effects on appetite, weight, mitochondrial function, renal and hepatic clearance, drug–drug interactions and the gut microbiome; recent evidence links medication burden and polypharmacy with altered gut microbial composition and function [83,84]. Therefore, in older adults, inflammatory biomarkers should be interpreted as signals arising from tissue aging, metabolic stress, inter-organ crosstalk, comorbidity burden, and therapeutic exposure rather than from metabolic dysfunction alone.

### 2.4. Why Inflammaging Matters for Metabolic Risk

Inflammaging matters for metabolic risk because it changes the biological meaning of conventional metabolic abnormalities. In younger or metabolically resilient individuals, modest nutrient excess, transient dysglycemia, or early lipid disturbance may be buffered by adipose tissue expandability, mitochondrial flexibility, endothelial repair, skeletal muscle glucose uptake and effective inflammatory resolution. In older adults, these compensatory systems are progressively weakened, so similar levels of abdominal adiposity, fasting glucose, triglycerides, or blood pressure may carry different biological risk depending on the underlying inflammatory state [2,85].

A central consequence is a lower threshold for insulin resistance. Chronic inflammatory signaling can interfere with insulin action across adipose tissue, skeletal muscle, and liver by activating stress-kinase pathways, promoting lipolysis, impairing glucose uptake, and increasing hepatic lipid accumulation [66,86]. Adipose tissue dysfunction is particularly important because impaired lipid storage, altered adipokine secretion, mitochondrial dysfunction and inflammation can drive systemic metabolic dysfunction and hepatic insulin resistance [11,87]. Thus, inflammation does not simply accompany metabolic dysfunction; it can reduce the metabolic reserve required to maintain glucose homeostasis [88].

Inflammaging also amplifies lipid and vascular injury. When inflammation impairs lipid storage and inter-organ metabolic coordination, excess fatty acids are more likely to be redirected toward liver, skeletal muscle and vascular tissues, promoting ectopic lipid deposition, oxidative stress, and endothelial dysfunction [86,87]. In parallel, inflammatory cytokines, microbial products and senescence-associated signals can reduce nitric oxide bioavailability, increase leukocyte adhesion, and accelerate arterial stiffness; these processes are central to vascular aging and cardiometabolic disease progression [89].

This concept has important implications for risk stratification. Population-level evidence shows that chronic low-grade inflammation is associated with higher risk and earlier onset of cardiometabolic multimorbidity in middle-aged and older adults [85]. Combined inflammatory biomarker patterns, including elevations in hsCRP and IL-6, have also been associated with adverse metabolic traits and cardiovascular mortality, supporting the use of inflammatory context to refine metabolic risk interpretation [90].

Having established inflammaging as the biological background of metabolic aging, the next question is how this inflammatory state reshapes the classical phenotype of metabolic syndrome. Rather than viewing central adiposity, dysglycemia, dyslipidemia, and hypertension as isolated diagnostic criteria, these abnormalities can be interpreted as clinical manifestations of immunometabolic stress.

Taken together, central adiposity and dysglycemia should be understood as overlapping but biologically distinct drivers of inflammatory-redox stress in aging. Central adiposity more directly reflects adipose expansion, immune-cell recruitment and altered adipokine signaling, whereas dysglycemia reflects impaired insulin action, glucotoxic stress, and metabolic inflexibility. These mechanisms provide the basis for reinterpreting conventional MetS components as biologically heterogeneous states in older adults.

## 3. From Metabolic Syndrome to Inflammatory Metabolic Phenotypes

**Figure 1:**
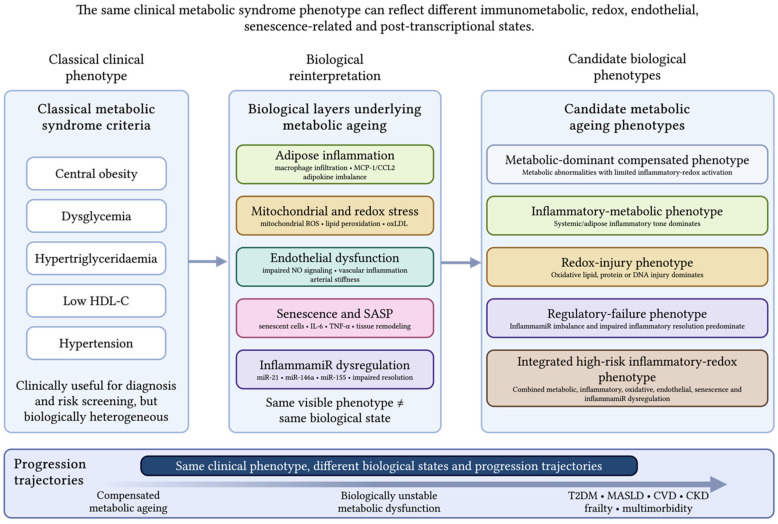
From metabolic syndrome criteria to metabolic aging phenotypes. Classical metabolic syndrome criteria remain clinically useful, but similar metabolic profiles in older adults may arise from distinct biological states. Central obesity, dysglycemia, hypertriglyceridemia, low HDL-C, and hypertension can be reinterpreted through layers of adipose inflammation, mitochondrial/redox stress, endothelial dysfunction, SASP activity, and inflammamiR dysregulation. These layers may define compensated, inflammatory-metabolic, redox-injury, regulatory-failure, and integrated high-risk inflammatory-redox phenotypes with different risks of progression to T2DM, MASLD, CVD, CKD, frailty, and multimorbidity. Abbreviations: CKD, chronic kidney disease; CVD, cardiovascular disease; DNA, deoxyribonucleic acid; HDL-C, high-density lipoprotein cholesterol; IL-6, interleukin-6; inflammamiR, inflammation-related microRNA; MASLD, metabolic dysfunction-associated steatotic liver disease; MCP-1/CCL2, monocyte chemoattractant protein-1/C-C motif chemokine ligand 2; miR, microRNA; NO, nitric oxide; oxLDL, oxidized low-density lipoprotein; ROS, reactive oxygen species; SASP, senescence-associated secretory phenotype; T2DM, type 2 diabetes mellitus; TNF-α, tumor necrosis factor-α. Created in BioRender. Asanova, A. (2026) https://BioRender.com/eqgluky (accessed 21 May 2026).

MetS is conventionally defined by the co-occurrence of central adiposity, dysglycemia, elevated blood pressure, hypertriglyceridemia, and low HDL-C. This diagnostic framework is clinically useful, but it only partially captures the biological heterogeneity of metabolic dysfunction in older adults. Building on the mechanisms described above, this section does not restate the inflammatory biology of adiposity and dysglycemia in detail. Instead, it considers how conventional MetS components may reflect partially distinct, although overlapping, clinical-biological states. Evidence from aging, obesity, and cardiometabolic research indicates that these clinical features can be accompanied by variable degrees of adipose tissue inflammation, altered insulin signaling, lipoprotein dysfunction, endothelial activation and inflammatory tissue remodeling (Figure 1) [31,32,33,36,37,38]. Therefore, individuals meeting similar metabolic syndrome criteria may differ in the biological processes contributing to their risk profile.

From this perspective, metabolic syndrome in later life can be considered not only as a clustering of cardiometabolic risk factors, but also as a clinical entry point for examining underlying immunometabolic and redox-related processes. This interpretation remains conceptual and does not imply that metabolic syndrome itself is a validated inflammatory phenotype. Rather, it suggests that conventional metabolic syndrome components may be biologically heterogeneous in older adults and may require complementary biomarker assessment in future studies.

Building on the mechanisms described above, this section does not restate the inflammatory biology of central adiposity and dysglycemia in detail. Instead, it reinterprets conventional MetS components as partially distinct, although overlapping, biological states in older adults. Central adiposity primarily reflects adipose expansion, immune-cell recruitment, and altered adipokine signaling, whereas dysglycemia reflects impaired insulin action, glucotoxic stress, and metabolic inflexibility. This distinction may help explain why older adults meeting similar MetS criteria can show different inflammatory, redox, and clinical trajectories.

This perspective reframes metabolic syndrome in later life not simply as a clustering of risk factors, but as a clinically visible manifestation of immunometabolic dysfunction. Central obesity may indicate expansion of visceral adipose tissue, but it may also signal adipocyte hypertrophy, macrophage recruitment, hypoxia, inflammasome activation, and senescence-associated secretory activity. Dysglycemia may reflect not only impaired insulin action, but also cytokine-mediated disruption of insulin signaling and β-cell stress. Similarly, hypertriglyceridemia and low HDL cholesterol may represent inflammatory lipid redistribution, oxidized or dysfunctional lipoproteins and impaired cholesterol efflux, whereas elevated blood pressure may reflect endothelial inflammation, oxidative injury, and vascular aging rather than hemodynamic load alone [6,33,34,35,38,91,92]. These tissue-specific sources and targets of inflammatory–redox–inflammamiR signaling are summarized in Table 2.

Inflammatory phenotyping may help identify older adults in whom metabolic syndrome is not merely a static diagnostic label, but a biologically active state-linking nutrient excess, adipose immune activation, endothelial dysfunction, and tissue remodeling. Circulating markers such as hs-CRP, IL-6, TNF-α, IL-1β, IL-18, MCP-1/CCL2, leptin, adiponectin, PAI-1, and galectin-3 may therefore refine the interpretation of waist circumference, fasting glucose, triglycerides, HDL cholesterol, and blood pressure by indicating systemic inflammation, adipose dysfunction, inflammasome activity, prothrombotic signaling, or inflammatory-fibrotic remodeling [31,32,33,36,37,38,41,92].

This distinction is particularly relevant in older adults with sarcopenic obesity, ectopic fat, vascular disease, or accumulating multimorbidity, in whom similar metabolic values may correspond to different risks of T2DM, MASLD, cardiovascular disease, frailty, and functional decline [31,32,37,80].

## 4. Redox Failure as the Amplifier of Metabolic Injury

Oxidative stress provides a mechanistic bridge between inflammaging and metabolic dysfunction because it links age-related mitochondrial impairment, chronic inflammatory signaling, and tissue-specific metabolic injury. In physiological conditions, reactive oxygen species (ROS) act as signaling molecules that participate in redox homeostasis, mitochondrial adaptation, and cellular stress responses. In metabolic aging, however, the key issue is not ROS production per se, but the loss of redox resilience—the declining capacity to use ROS as adaptive signals while preventing sustained oxidative molecular damage. When ROS production exceeds antioxidant, proteostatic, and repair capacity, oxidative stress promotes lipid peroxidation, protein oxidation, DNA damage, endothelial dysfunction, and impaired insulin signaling [93]. Aging increases vulnerability to this loss of redox resilience through mitochondrial dysfunction, reduced antioxidant capacity, impaired proteostasis, cellular senescence and chronic activation of inflammatory pathways [5]. Conversely, inflammatory cytokines, immune-cell activation and metabolic danger signals can stimulate ROS production, creating a reciprocal inflammatory–redox loop in which oxidative stress amplifies inflammation and inflammation reinforces oxidative injury [5,94]. In metabolic syndrome, obesity, and T2DM, this loop is particularly relevant because nutrient excess, lipotoxicity, hyperglycemia and mitochondrial ROS converge to overwhelm redox resilience, impair insulin-sensitive tissues, promote vascular dysfunction and accelerate cardiometabolic complications [93,95]. Therefore, oxidative stress should not be viewed as a downstream by-product of metabolic disease alone, but as an active mediator that converts inflammaging into insulin resistance, endothelial injury, dyslipidemia-related damage, and progressive metabolic organ dysfunction.

### 4.1. Sources of Oxidative Stress in Metabolic Aging

Oxidative stress in metabolic aging reflects a shift in redox homeostasis rather than a single source of ROS. In metabolically resilient tissues, ROS generated during nutrient oxidation, immune activation, or vascular signaling can be buffered by mitochondrial quality control, antioxidant enzymes, and adaptive redox responses. With aging, this buffering becomes less stable: mitochondrial efficiency declines, inflammatory signaling persists, antioxidant coordination weakens, and metabolic substrates are increasingly redirected toward ectopic tissues. The result is a feed-forward redox network in which mitochondria, NADPH oxidases, hyperglycemia, lipid overload, inflammation, and endothelial dysfunction reinforce one another [5,93,96].

Mitochondrial dysfunction is a central contributor. Although mitochondria normally generate ROS during oxidative phosphorylation, aging, nutrient overload, and impaired mitophagy increase electron leakage and reduce energetic flexibility. In insulin-sensitive tissues, mitochondrial ROS can impair insulin signaling, promote lipid peroxidation, and activate inflammatory pathways [5,95,97]. Human experimental evidence supports this mechanism: reducing mitochondrial oxidative burden alleviated lipid-induced skeletal muscle insulin resistance, directly implicating mitochondrial oxidants in metabolic dysfunction [98]. In the liver, dysglycemia, and intrahepatic lipid accumulation are closely coupled to altered hepatic oxidative capacity during insulin resistance, linking substrate overload with mitochondrial stress [99].

NADPH oxidases provide a second major ROS-generating system. In obesity, diabetes, and MetS, NOX-derived ROS contribute to adipose inflammation, vascular oxidative stress, and impaired insulin action [100,101,102]. Experimental data show that adipocyte-specific NOX4 deficiency delays obesity-induced insulin resistance and attenuates adipose inflammation, supporting a causal role for adipocyte NOX signaling [103]. Other studies connect NOX4 with adipocyte differentiation, lipid accumulation, and oxidative injury from adipose-derived signals [104,105]. However, NOX biology is context-dependent, as skeletal muscle NOX4 can also support adaptive exercise responses, reinforcing that redox signaling is not uniformly pathological [106].

Hyperglycemia and lipid overload further amplify redox stress. Hyperglycemia increases mitochondrial ROS, protein glycation, polyol-pathway flux, protein kinase C activation, and endothelial nitric oxide synthase uncoupling; in vascular cells, high glucose increases ROS production and endothelin-1 expression [107]. Oxidative stress also reduces nitric oxide bioavailability and contributes to microvascular and macrovascular complications [108]. Excess fatty acids, triglyceride-rich lipoproteins, and ectopic lipid deposition increase mitochondrial substrate pressure, endoplasmic reticulum stress, ceramide and diacylglycerol accumulation, and inflammatory signaling. In skeletal muscle, lipotoxicity interacts with mitochondrial dysfunction to impair insulin sensitivity [98,109], whereas in liver and pancreas, chronic fatty-acid and glucose exposure promotes oxidative stress, inflammation, and β-cell dysfunction [99,110]. Fatty acids also alter endothelial redox state, linking dyslipidemia to vascular oxidative injury [111].

Chronic inflammation and endothelial dysfunction close this amplification loop. Activated macrophages, senescent cells, and cytokine-stimulated endothelial cells generate ROS through mitochondrial, NOX-dependent, and inflammatory enzyme pathways; in turn, ROS activate NF-κB and inflammasome-related signaling [12,93]. Endothelial oxidative stress reduces nitric oxide bioavailability, increases leukocyte adhesion, and contributes to arterial stiffness and vascular aging [68,89]. Oxidative stress intensifies further when antioxidant defenses become insufficient, including glutathione pathways, superoxide dismutases, catalase, and NRF2-mediated transcriptional responses [112,113]. Population studies using oxidative balance scores suggest that lower pro-oxidant burden and higher antioxidant exposure are associated with lower MetS risk and better outcomes in middle-aged or older adults [114,115,116]. Together, these findings position oxidative stress as both increased ROS generation and declining redox resilience between inflammaging and metabolic injury.

### 4.2. Oxidative Damage, Antioxidant Failure, and Inflammatory Amplification

Oxidative stress becomes metabolically consequential when ROS are no longer confined to adaptive signaling, but instead modify lipids, proteins, and redox-sensitive inflammatory pathways. Lipid peroxidation is one of the earliest and most biologically active consequences of this shift. Peroxidation of polyunsaturated fatty acids generates products such as F2-isoprostanes, malondialdehyde (MDA), 4-hydroxynonenal (4-HNE), and oxidized low-density lipoprotein (oxLDL), which are not merely passive markers of oxidative injury but can actively propagate metabolic and vascular dysfunction (Table 3). F2-isoprostanes are widely regarded as robust systemic indicators of lipid peroxidation, whereas MDA and thiobarbituric acid-reactive substances are more accessible but less specific. 4-HNE is particularly relevant because it forms adducts with proteins and nucleic acids, alters mitochondrial and insulin-signaling pathways, and has been linked to insulin resistance and endothelial dysfunction; recent reviews emphasize its role as a bioactive lipid-peroxidation mediator rather than only a degradation product [117]. OxLDL provides a direct vascular link because oxidative modification of low-density lipoprotein promotes endothelial activation, foam-cell formation, inflammatory signaling, and atherogenesis.

Protein oxidation represents a second layer of redox-mediated metabolic injury. Protein carbonyls and advanced oxidation protein products (AOPPs) reflect cumulative oxidative modification of circulating and tissue proteins. These modifications can impair enzyme activity, receptor signaling, transporter function, extracellular-matrix integrity and mitochondrial protein function, thereby translating oxidative stress into cellular dysfunction. AOPPs are especially relevant because they integrate oxidative and inflammatory biology: they are enriched in chronic inflammatory and metabolic disorders, including diabetes, chronic kidney disease, obesity, and MetS, and experimental data show that AOPPs can promote pancreatic β-cell apoptosis through NADPH oxidase-dependent superoxide generation [123]. Recent clinical work also links AOPPs with altered lipoprotein subclasses, supporting their relevance to the intersection of protein oxidation, dyslipidemia, and cardiometabolic risk [124]. Thus, protein oxidation can both reflect and reinforce metabolic injury by damaging proteins that maintain insulin responsiveness, endothelial homeostasis, and lipid transport.

The pathological significance of lipid and protein oxidation depends partly on the capacity of antioxidant defense systems to buffer redox stress. Superoxide dismutases convert superoxide into hydrogen peroxide, catalase and glutathione peroxidases detoxify hydrogen peroxide and lipid hydroperoxides, and the reduced-to-oxidized glutathione ratio reflects intracellular redox reserve. These systems are coordinated partly through nuclear factor erythroid 2-related factor 2 (NRF2), a transcriptional regulator of antioxidant and cytoprotective responses. Contemporary reviews describe antioxidant defense as a multilayered system involving superoxide dismutase, catalase, glutathione peroxidase and glutathione-dependent pathways [125]. However, aging and metabolic disease can uncouple this defense network: increased antioxidant enzyme activity may indicate compensation, whereas reduced activity or depleted glutathione may indicate exhaustion. Altered NRF2 signaling in systemic redox imbalance further suggests that failure of transcriptional antioxidant adaptation can contribute to non-communicable diseases and metabolic vulnerability [113]. Consequently, antioxidant markers should be interpreted together with oxidative-damage products rather than as isolated indicators of “protection” (Table 3).

Oxidative stress and inflammation then become self-reinforcing. ROS can activate redox-sensitive inflammatory pathways, including NF-κB, mitogen-activated protein kinase signaling and inflammasome pathways; recent reviews specifically highlight ROS-driven NF-κB activation and NLRP3 inflammasome regulation in metabolic and inflammatory disease contexts [126,127]. Conversely, inflammatory cytokines, activated macrophages, neutrophils, and endothelial cells stimulate mitochondrial ROS production, NADPH oxidase activity and myeloperoxidase-dependent oxidant generation. This reciprocal loop helps explain why oxidative stress intensifies insulin resistance, endothelial dysfunction, lipid modification, and tissue remodeling, while chronic inflammation sustains the oxidant burden. In metabolic aging, therefore, oxidative biomarkers do not simply measure accumulated damage; they identify an active inflammatory-redox state that can connect inflammaging with progressive metabolic injury and prepare the mechanistic ground for cytokine-miRNA-oxidative stress feedback loops.

## 5. InflammamiRs as Post-Transcriptional Regulators of Inflammatory-Redox Adaptation

miRNAs add a post-transcriptional regulatory layer to the inflammatory–redox model of metabolic dysfunction. These small non-coding RNAs regulate gene expression mainly by binding target messenger RNAs and modulating transcript stability or translation, thereby allowing one miRNA to influence multiple pathways involved in immune activation, mitochondrial stress, oxidative signaling, adipogenesis, insulin action, and vascular function [18,128]. In biomarker research, miRNAs are attractive because they are detectable in serum and plasma, can be protected from degradation through association with extracellular vesicles, Argonaute proteins, or lipoproteins, and may partly reflect tissue-to-blood communication during metabolic stress [129,130].

Within this broader miRNA landscape, “inflammamiRs” refer to miRNAs involved in inflammatory activation, immune-cell regulation, cytokine signaling and inflammatory resolution [131]. The concept is particularly relevant to metabolic aging (Table 4) because miR-21, miR-146a and miR-155 sit at the intersection of NF-κB-related signaling, inflammasome regulation, cytokine responses, oxidative stress and cardiometabolic disease biology [19,132].

Among inflammation-related miRNAs, miR-21-5p, miR-146a-5p, and miR-155-5p are especially relevant to metabolic aging because they occupy distinct but interconnected positions within inflammatory signaling. miR-21-5p is a stress-responsive and remodeling-associated miRNA that appears in cardiometabolic, obesity, and diabetes-related signatures, although its meaning is context-dependent and may reflect injury, repair, fibrosis, or chronic inflammatory activation. Recent circulating-miRNA reviews identify miR-21 and miR-146a as candidate obesity-related inflammatory biomarkers, supporting their potential as blood-accessible signals of metabolic inflammation [130]. miR-146a-5p is best understood as a compensatory brake on innate immune activation. It is induced by inflammatory signaling and restrains TLR and IL-1 receptor pathways through IRAK1 and TRAF6 repression, thereby limiting downstream NF-κB activity and cytokine production [148,149]. In aging, altered circulating miR-146a-5p may therefore indicate impaired inflammatory feedback rather than simply “low inflammation,” particularly because it has been proposed as an inflammaging-related miRNA and varies with age and diabetes status [19,20].

miR-155-5p represents the more pro-inflammatory arm of the inflammamiR network. It is linked to macrophage activation, cytokine production, and immune-metabolic reprogramming, and cytokine-miRNA reviews place miR-155 within dysregulated networks relevant to obesity, T2DM, and cardiovascular disease [132]. In metabolic tissues, miR-155 may indicate active inflammatory pressure, especially when interpreted with cytokines, adipokines, and insulin-resistance indices. Mechanistic evidence shows that adipose tissue macrophage-derived exosomal miR-155 can impair insulin sensitivity by targeting PPARγ, supporting its role in obesity-associated inflammatory insulin resistance [137]. More broadly, extracellular vesicle-derived miRNAs from adipose tissue macrophages and other metabolic tissues can influence insulin sensitivity, β-cell adaptation, and inter-organ metabolic communication [150,151]. Reviews of plasmatic and exosomal miRNAs in oxidative stress-related metabolic diseases further support separating total circulating miRNA signals from EV-enriched miRNA communication [152]. However, circulating miR-155 should not be treated as a stand-alone biomarker because circulating and extracellular-vesicle miRNA profiles depend on tissue source, vesicle release, disease stage, and metabolic context [153,154].

Other inflammamiRs may be more informative for tissue-specific complications. miR-223-3p is myeloid-enriched and may be most useful when metabolic dysfunction is dominated by macrophage–inflammasome biology, consistent with the role of NLRP3 activation in adipose inflammation, insulin resistance, and T2DM [155]. miR-29 family members and miR-34a-5p are more closely aligned with diabetic tissue injury, extracellular-matrix remodeling, mitochondrial stress, and hepatic metabolic dysfunction. Reviews of miRNAs in MASLD identify miR-34a and miR-29a as regulators of steatosis, inflammation, fibrosis, and metabolic stress, with miR-34a particularly linked to SIRT1-related metabolic regulation [156]. These miRNAs may therefore better indicate organ-level metabolic injury than generic inflammation.

Endothelial and vascular inflammamiRs form another axis. miR-126-3p/5p is enriched in endothelial biology and is repeatedly implicated in angiogenesis, endothelial repair, and diabetic vascular complications; recent reviews associate dysregulated miR-126 with impaired angiogenesis, endothelial dysfunction, and accelerated diabetic complications [157]. Clinical work combining circulating miR-21, circulating miR-126, redox status and inflammatory conditions further supports the concept that vascular damage in T2DM is better captured by integrated miRNA–redox–inflammatory profiles than by a single biomarker [158]. The miR-200 family, especially miR-200c, provides a complementary redox-sensitive vascular signal linked to oxidative stress-associated endothelial dysfunction in diabetes [159]. Consistent with this multidomain interpretation, integrated models combining microRNA expression, lipid profiles, and oxidative-stress markers have been proposed for predicting macrovascular complications in T2DM [160]. By contrast, miR-9-5p and miR-132-3p remain more exploratory in metabolic aging. They are biologically plausible because they intersect with cytokine, inflammasome, neuronal-immune, and cardiometabolic signaling networks, but their evidence base is narrower and more context-specific than that of miR-21, miR-146a, miR-155, miR-223, miR-34a, or miR-126 [136,140,161]. These latter miRNAs are more consistently represented across inflammaging, obesity, diabetes, and the cardiometabolic disease literature [132].

Overall, inflammamiRs should be interpreted as regulatory fingerprints of inflammatory-redox metabolic states rather than isolated diagnostic markers. Their greatest value is likely to come from multi-miRNA panels integrated with cytokines, oxidative-stress biomarkers, and conventional metabolic markers. In this framework, miR-21, miR-146a, and miR-155 may indicate inflammatory activation and resolution capacity; miR-223 may reflect myeloid–inflammasome activity; miR-29 and miR-34a may capture diabetic and hepatic tissue injury; and miR-126 and miR-200 family members may identify vascular involvement.

A major limitation of circulating inflammamiRs is biological ambiguity. Increased plasma miR-21, miR-146a, or miR-155 may reflect leukocyte activation, adipose-tissue inflammation, endothelial injury, platelet contamination, extracellular-vesicle release, or altered clearance. Direction of change also does not necessarily indicate pathogenicity: miR-146a may rise as compensatory feedback or fall as a marker of failed inflammatory resolution. Therefore, inflammamiRs should not be interpreted as disease-specific biomarkers unless contextualized by cell source, EV fraction, hemolysis control, normalization strategy, and concurrent cytokine/redox markers. Future studies should distinguish total circulating miRNAs from extracellular-vesicle-enriched, lipoprotein-associated, and Argonaute-bound fractions because these compartments may carry different biological meanings.

## 6. Inflammaging–Redox–InflammamiR Axis

The Inflammaging–Redox–InflammamiR Axis proposed here should be interpreted as a conceptual synthesis of currently available evidence rather than as a validated mechanistic or clinical model. Although inflammation, oxidative stress, mitochondrial dysfunction, and inflammation-related miRNAs have each been linked to metabolic aging, direct evidence that these processes operate as a unified axis in humans remains limited. Therefore, the framework is intended to organize overlapping biological observations and identify testable hypotheses for future longitudinal and interventional studies.

**Figure 2:**
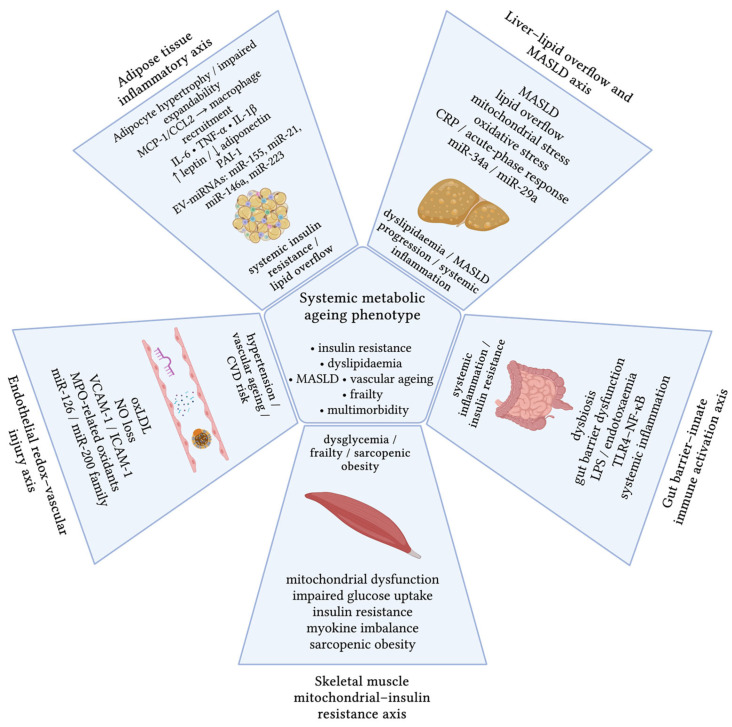
Tissue-specific inflammatory–redox–inflammamiR axes in metabolic aging. Note: Tissue-specific inflammatory, redox, and miRNA-related axes provide major sources and targets of systemic metabolic aging. Radial panels represent conceptual tissue-specific axes that converge on the central systemic metabolic aging phenotype. Upward and downward arrows indicate the expected relative direction of change in the indicated biomarker signals during inflammatory or immunometabolic activation; they do not represent diagnostic thresholds, validated clinical cutoffs, or quantitative effect sizes. In adipose tissue, impaired adipose expandability, adipocyte hypertrophy, and macrophage recruitment promote MCP-1/CCL2 signaling, cytokine production, leptin/adiponectin imbalance, PAI-1 release, and extracellular-vesicle miRNA communication. In the liver, adipose-derived lipid overflow contributes to MASLD, mitochondrial stress, oxidative injury, acute-phase signaling and miR-34a/miR-29a-related stress responses. In skeletal muscle, ageing, inactivity, lipid infiltration and mitochondrial dysfunction impair glucose uptake, reduce metabolic flexibility, and alter myokine signaling, thereby promoting insulin resistance, dysglycemia and sarcopenic obesity. In the endothelium, oxLDL, ROS and inflammatory mediators reduce nitric oxide bioavailability and promote adhesion molecule expression, vascular inflammation, and endothelial repair failure, with miR-126 and the miR-200 family representing vascular miRNA signals. In the gut, dysbiosis and impaired barrier function facilitate LPS translocation and TLR4–NF-κB activation, contributing to systemic low-grade inflammation and insulin resistance. These tissue axes converge on systemic metabolic aging phenotypes, including insulin resistance, dyslipidemia, MASLD, vascular aging, frailty and multimorbidity. Abbreviations: CRP, C-reactive protein; CVD, cardiovascular disease; EV, extracellular vesicle; ICAM-1, intercellular adhesion molecule 1; IL, interleukin; LPS, lipopolysaccharide; MASLD, metabolic dysfunction-associated steatotic liver disease; MCP-1/CCL2, monocyte chemoattractant protein-1/C-C motif chemokine ligand 2; miR, microRNA; miRNA, microRNA; MPO, myeloperoxidase; NF-κB, nuclear factor-κB; NO, nitric oxide; oxLDL, oxidized low-density lipoprotein; PAI-1, plasminogen activator inhibitor-1; ROS, reactive oxygen species; T2DM, type 2 diabetes mellitus; TLR4, Toll-like receptor 4; TNF-α, tumor necrosis factor-α; VCAM-1, vascular cell adhesion molecule 1. Created in BioRender. Asanova, A. (2026) https://BioRender.com/ilwbgbk (accessed 21 May 2026).

Single-pathway explanations are insufficient for age-associated metabolic dysfunction because cytokines, ROS and miRNAs may operate as interdependent regulatory circuits rather than independent biomarker classes. Cytokines such as IL-6, TNF-α and IL-1β can alter miRNA expression through NF-κB, JAK/STAT and inflammasome-linked pathways; in turn, miRNAs regulate cytokine production, immune-cell activation, macrophage polarization, and inflammatory resolution. Dysregulated cytokine–miRNA networks have been implicated in obesity, T2DM and cardiovascular disease through effects on insulin sensitivity, lipid metabolism and inflammatory signaling [132]. Similarly, inflammation-related miRNAs are increasingly recognized as regulators of inflammatory pathway activity and potential therapeutic targets in obesity, diabetes, cardiovascular disease, and MASLD [128,131]. Thus, a single cytokine or miRNA rarely captures the regulatory state of metabolic aging; each represents only one node within a dynamic, tissue-distributed network (Figure 2).

**Figure 3:**
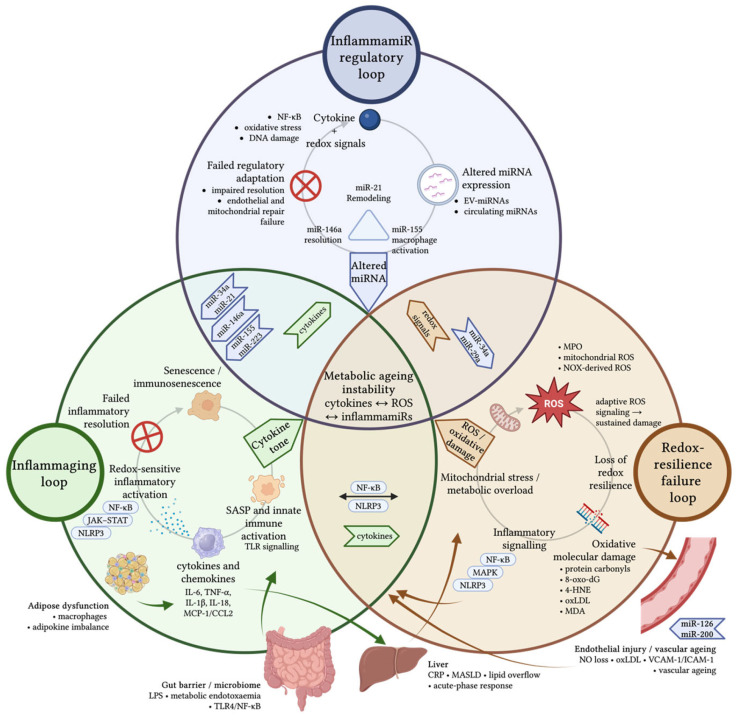
The Inflammaging–Redox–InflammamiR feedback-loop network in metabolic aging. Note: The figure presents age-associated metabolic dysfunction as three interlocking feedback loops rather than a linear pathway. The inflammaging loop links senescence, immunosenescence, adipose dysfunction and gut-derived innate immune stimulation with SASP activity, cytokine production including IL-6, TNF-α, IL-1β, IL-18 and MCP-1/CCL2, and NF-κB, JAK–STAT, JNK/p38 MAPK and NLRP3 signaling. The redox-resilience failure loop links mitochondrial stress, metabolic overload, NOX-derived ROS and MPO-related oxidants with oxidative molecular damage including lipid peroxidation, protein oxidation and oxidative DNA damage and redox-sensitive inflammatory activation. The inflammamiR regulatory loop shows how cytokine and redox signals alter circulating and EV-associated miRNAs. miR-21, miR-146a and miR-155 form a regulatory triangle connecting tissue remodeling, inflammatory resolution and macrophage activation, while miR-223, miR-29a, miR-34a, miR-126 and the miR-200 family extend the axis to myeloid–inflammasome, mitochondrial–senescence and endothelial-vascular pathways. Together, these loops converge on metabolic aging instability through reciprocal reinforcement of cytokines, ROS, and inflammamiRs. Abbreviations: 4-HNE, 4-hydroxynonenal; 8-oxo-dG, 8-oxo-2′-deoxyguanosine; CCL2, C-C motif chemokine ligand 2; CRP, C-reactive protein; EV, extracellular vesicle; ICAM-1, intercellular adhesion molecule 1; IL, interleukin; JAK–STAT, Janus kinase–signal transducer and activator of transcription; LPS, lipopolysaccharide; MAPK, mitogen-activated protein kinase; MCP-1, monocyte chemoattractant protein-1; MDA, malondialdehyde; miR, microRNA; miRNA, microRNA; MPO, myeloperoxidase; NF-κB, nuclear factor-κB; NLRP3, nucleotide-binding oligomerization domain-like receptor family pyrin domain-containing 3; NO, nitric oxide; NOX, NADPH oxidase; oxLDL, oxidized low-density lipoprotein; ROS, reactive oxygen species; SASP, senescence-associated secretory phenotype; TLR, Toll-like receptor; TLR4, Toll-like receptor 4; TNF-α, tumor necrosis factor-α; VCAM-1, vascular cell adhesion molecule 1. Created in BioRender. Asanova, A. (2026) https://BioRender.com/fl066km (accessed 21 May 2026).

Oxidative stress adds another layer of feedback. ROS activate redox-sensitive inflammatory pathways, including NF-κB and NLRP3 inflammasome signaling, whereas inflammatory cytokines, activated macrophages and endothelial cells stimulate mitochondrial ROS production, NADPH oxidase activity and myeloperoxidase-dependent oxidant generation [126,162]. Oxidative stress can also reshape miRNA expression through redox-sensitive transcription factors, DNA-damage responses, mitochondrial stress and lipid-peroxidation products; miRNAs then regulate antioxidant defense, mitochondrial function, endothelial repair and inflammatory ROS production [128,163]. Therefore, metabolic dysfunction should not be viewed as a linear sequence from inflammation to oxidative damage to insulin resistance, but as a self-amplifying inflammatory-redox-post-transcriptional system that may explain divergent clinical trajectories among older adults with similar glucose, lipid, or anthropometric profiles (Figure 3).

The Inflammaging–Redox–InflammamiR Axis can be conceptualized as the regulatory interface through which biological aging is converted into metabolic injury. The major feedback-loop components of this axis are summarized in Table 5. Aging tissues accumulate immune senescence, cellular senescence, mitochondrial dysfunction, impaired proteostasis, and altered intercellular communication, which are interconnected features of aging biology [4,5,164]. Senescent cells release cytokines and chemokines, dysfunctional mitochondria increase ROS, and chronic metabolic stress reshapes post-transcriptional regulation through inflammation-sensitive miRNAs [4,128]. In this framework, aging hallmarks provide the upstream biological substrate, whereas the phenotypic hallmarks of MetS—central adiposity, dysglycemia, hypertriglyceridemia, reduced HDL-C and elevated blood pressure—represent downstream clinical expressions of disrupted inflammatory-redox-miRNA regulation.

At the upstream level, cytokines and ROS form a tightly coupled inflammatory-redox circuit. IL-6, TNF-α, and IL-1β can increase mitochondrial stress, NADPH oxidase activity, and antioxidant-system exhaustion, whereas ROS activate redox-sensitive pathways such as NF-κB, JNK, p38 mitogen-activated protein kinase and inflammasome signaling [126,162,165]. Once established, this cytokine-ROS loop can promote impaired insulin signaling, adipose inflammation, endothelial dysfunction, and hepatic lipid stress, linking aging biology to insulin resistance, dyslipidemia, and vascular injury [126,132].

InflammamiRs add a post-transcriptional regulatory layer. Cytokine signaling can alter miR-21, miR-146a and miR-155 expression, while these miRNAs feed back onto inflammatory pathways [128,163]. miR-146a represents a compensatory arm because it is induced by inflammatory signaling and restrains TLR and IL-1 receptor pathways through IRAK1 and TRAF6 suppression [148,149,166]. By contrast, miR-155 is more closely aligned with inflammatory macrophage activation and impaired insulin sensitivity, with original evidence showing that adipose tissue macrophage-derived exosomal miRNAs, including miR-155, modulate insulin sensitivity [137]. miR-21 is linked to stress adaptation, tissue remodeling and inflammatory–fibrotic responses, especially when metabolic injury becomes chronic rather than transient [128,163].

ROS also regulate miRNA biology. Oxidative stress can reshape miRNA expression through redox-sensitive transcription factors, DNA-damage responses, mitochondrial stress and lipid-peroxidation products [151,163]. This creates an additional feedback layer in which oxidative injury induces miRNAs that regulate mitochondrial function, antioxidant defense, endothelial repair, and inflammatory ROS production. For example, miR-34a may reinforce mitochondrial and senescence-associated stress through SIRT1-related pathways [167]; miR-29a may participate in high-glucose mitochondrial and inflammatory tissue responses [141,142]; and endothelial miR-126 dysregulation may weaken vascular repair under oxidative endothelial injury [157,168]. Thus, inflammamiRs are not merely downstream consequences of oxidative stress; they can influence whether redox stress is buffered, propagated, or translated into tissue dysfunction.

This axis is especially important in metabolic tissues. In visceral adipose tissue, macrophage-derived and adipocyte-derived signals connect cytokine production, ROS generation and extracellular-vesicle miRNA transfer [137,169]. In the liver, oxidative stress and inflammatory signaling interact with miRNAs involved in lipid accumulation, mitochondrial stress, and hepatocellular injury [128]. In skeletal muscle, inflammatory and redox stress impair insulin-stimulated glucose uptake, whereas in the endothelium ROS-sensitive miRNAs contribute to vascular inflammation, impaired nitric oxide signaling and reduced repair capacity [157,168]. These tissue-specific loops converge systemically, allowing local adipose, hepatic, or vascular inflammation to become a broader metabolic phenotype.

The value of the Inflammaging–Redox–InflammamiR Axis is that it explains how aging hallmarks are translated into the phenotypic hallmarks of metabolic dysfunction. Age-associated metabolic dysfunction emerges from a self-reinforcing network in which immune senescence and senescence-associated secretory activity increase cytokine exposure; cytokines and metabolic stress promote ROS generation and oxidative molecular damage; oxidative stress alters inflammamiR expression; and inflammamiRs regulate inflammatory resolution, macrophage activation, mitochondrial resilience, and endothelial function [128,132]. Conceptually, miR-21, miR-155 and miR-146a form a regulatory triangle linking inflammatory activation, redox injury, and failed metabolic adaptation. As these loops intensify, the phenotype shifts from compensated metabolic aging toward central adiposity-driven inflammation, insulin resistance, dyslipidemia, endothelial dysfunction, hepatic steatosis, vascular aging, and progressive cardiometabolic disease. This feedback-loop model provides a mechanistic rationale for moving beyond isolated biomarkers. The next translational challenge is to organize clinical, metabolic, inflammatory, oxidative, and inflammamiR markers into interpretable biomarker tiers and patient phenotypes that can support risk stratification and monitoring.

It is important to emphasize that the Inflammaging–Redox–InflammamiR Axis should be interpreted as a working conceptual model rather than as a fully established pathophysiological pathway. Substantial evidence supports pairwise interactions among chronic low-grade inflammation, oxidative stress, mitochondrial dysfunction, adipose tissue inflammation, endothelial injury, and miRNA-mediated regulation. However, direct evidence showing that these components operate as a unified and reproducible axis in human metabolic aging remains limited. Therefore, the term “axis” is used here as a heuristic framework to organize biologically related processes and generate testable hypotheses, rather than to imply a validated causal sequence. Future longitudinal, tissue-informed and intervention-based studies will be required to determine whether inflammatory, redox, and inflammamiR signals improve prediction beyond conventional metabolic markers and whether they identify reproducible biological subgroups.

## 7. Integrated Biomarker Tiers and Candidate Risk Phenotypes

The biomarker tiers and risk phenotypes described in this section are proposed as author-derived, hypothesis-generating constructs. They should not be interpreted as established diagnostic categories or validated clinical phenotypes. Rather, they are intended to illustrate how conventional metabolic markers might be combined with inflammatory, redox, mitochondrial, senescence-related, and inflammamiR markers in future studies. Their clinical value will require prospective validation, standardized assays, external replication, and evidence that they improve prediction beyond established metabolic and cardiometabolic risk criteria.

### 7.1. Why Single Biomarkers Are Insufficient

The feedback-loop model described above has direct implications for risk stratification, but these implications remain conceptual and require empirical testing. Age-associated metabolic dysfunction is unlikely to be resolved by any single inflammatory, oxidative-stress or miRNA marker because it reflects the convergence of metabolic load, adipose dysfunction, inflammatory tone, redox injury, endothelial involvement, tissue remodeling and post-transcriptional regulation. Conventional markers define the observable metabolic phenotype, but they do not indicate whether that phenotype is inflammatory, redox-dominant, regulatory-dysregulated, or already progressing toward tissue injury.

Each biomarker class adds a different layer of information but also has specific constraints. hs-CRP is clinically accessible but non-specific [170,171]; cytokines such as IL-6, TNF-α and IL-1β are mechanistically informative but dynamic and context-dependent [172,173,174,175]; oxidative-stress biomarkers are sensitive to assay selection and pre-analytical handling [176]; and circulating miRNAs are influenced by hemolysis, blood-cell contamination, extracellular-vesicle fraction, normalization strategy, medication exposure, and tissue source [163,177,178]. These limitations support a multidomain rather than single-marker approach.

Population-based evidence shows that chronic low-grade inflammation is associated with higher risk and earlier onset of cardiometabolic multimorbidity in middle-aged and older adults [85]. Studies integrating inflammatory and oxidative markers further indicate that MetS components are accompanied by heterogeneous inflammatory-redox profiles rather than a uniform biomarker pattern [179]. In multimorbidity research, systematic reviews similarly emphasize that longitudinal, multidomain and multi-omics approaches are needed to capture biological heterogeneity in aging populations [180,181]. The relevant translational question is therefore how clinical, metabolic, inflammatory, oxidative and miRNA layers can be integrated into interpretable risk models for patient phenotyping, monitoring and predictive classification [178,182].

### 7.2. Proposed Tiered Biomarker Framework

**Figure 4:**
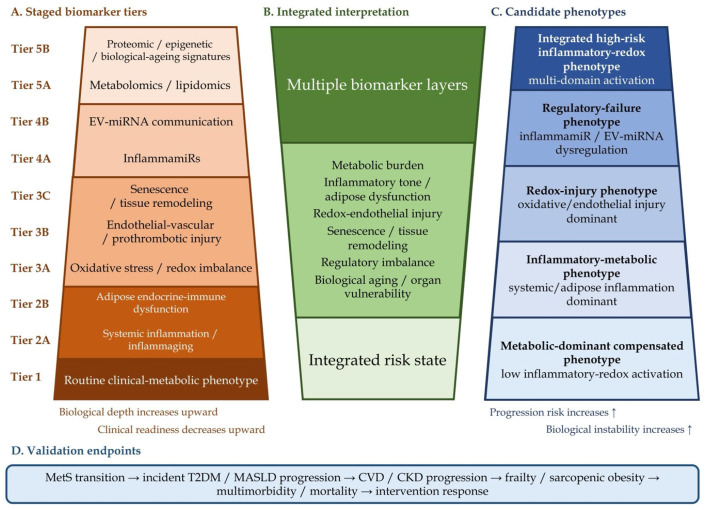
Proposed biomarker tiers and clinical phenotypes for metabolic aging. Panel (**A**) shows the staged biomarker framework. Tier 1 represents the routine clinical-metabolic phenotype, whereas progressively higher tiers add systemic inflammation and inflammaging, adipose endocrine-immune dysfunction, oxidative-redox imbalance, endothelial-vascular and prothrombotic injury, senescence-associated tissue remodeling, inflammamiRs, EV-miRNA communication, and multi-omic or biological-aging signatures. Biological depth increases upward, whereas immediate clinical readiness decreases upward. Panel (**B**) illustrates how multiple biomarker layers can be integrated into broader biological axes, including metabolic burden, inflammatory tone, adipose dysfunction, redox-endothelial injury, senescence-related remodeling, regulatory imbalance and biological aging or organ vulnerability. These axes converge into an integrated risk state. Panel (**C**) translates the integrated biomarker profile into candidate metabolic aging phenotypes ordered by increasing progression risk and biological instability, from the metabolic-dominant compensated phenotype to the integrated high-risk inflammatory-redox phenotype. Panel (**D**) summarizes the clinical endpoints needed to validate the framework, including transition to MetS, incident T2DM, MASLD progression, CVD or CKD progression, frailty or sarcopenic obesity, multimorbidity, mortality and intervention response. The figure is conceptual; biomarker tiers and phenotypes differ in evidentiary maturity and should not be interpreted as equally validated. These phenotypes are proposed as hypothesis-generating constructs rather than validated diagnostic categories. Abbreviations: CKD, chronic kidney disease; CVD, cardiovascular disease; EV, extracellular vesicle; MASLD, metabolic dysfunction-associated steatotic liver disease; MetS, metabolic syndrome; miRNA, microRNA; T2DM, type 2 diabetes mellitus.

We propose a tiered biomarker framework that organizes markers according to clinical accessibility, biological depth, and translational readiness (Table 6). This tiered structure should be understood as a staged research-to-clinic model: Tier 1 defines the routine clinical-metabolic phenotype, and selected Tier 2 markers may support near-term clinical enrichment, whereas Tiers 3–5 are currently best positioned for mechanistic phenotyping, cohort discovery and prediction-model development. Higher-tier biomarkers are therefore not inherently superior; rather, they provide progressively deeper biological resolution of inflammation, adipose dysfunction, redox injury, endothelial and senescence-related damage, post-transcriptional regulation, inter-tissue communication, and multi-omic aging signatures. The aim is to clarify what each layer adds to risk stratification, longitudinal monitoring and predictive model development, while recognizing that clinical translation requires validation and interpretability [183]. To connect biomarker biology with clinical translation, we propose a staged tier-to-phenotype framework that links routine metabolic markers, inflammatory and redox biomarkers, inflammamiRs and multi-omic signatures to candidate progression-risk phenotypes (Figure 4).

Tier 1 represents the routine clinical-metabolic phenotype. These markers are closest to current practice and include anthropometry, blood pressure, glycemic markers, insulin resistance indices, lipid measures, liver enzymes, and renal function. Their strength is feasibility: they are inexpensive, standardized, and already embedded in screening, diagnosis and longitudinal monitoring. However, they mainly describe the phenotypic expression of metabolic dysfunction rather than its biological drivers. For example, waist circumference indicates adiposity, but not whether adipose tissue is inflamed; triglycerides indicate lipid burden, but not whether dyslipidemia is accompanied by oxidative lipid modification; and HbA1c captures glycemic exposure, but not the inflammatory or mitochondrial state that may accelerate tissue injury [6,184]. Thus, Tier 1 provides the necessary clinical frame, but cannot alone distinguish stable metabolic aging from biologically active inflammatory-redox metabolic dysfunction.

Tiers 2 and 3 add mechanistic phenotyping. Tier 2 captures systemic inflammation, inflammaging, and adipose endocrine-immune dysfunction. These markers help interpret whether a conventional metabolic phenotype is accompanied by chronic inflammatory tone, innate immune activation, adipose macrophage recruitment, impaired adipokine balance or prothrombotic adipose dysfunction [31,32,37,38]. Tier 3 captures downstream injury biology: oxidative damage, antioxidant reserve, endothelial activation, vascular aging, prothrombotic signaling and tissue-remodeling, or senescence-associated signals. This tier is particularly important because oxidative and endothelial markers can identify whether metabolic risk has progressed from biochemical abnormality to molecular damage and vascular involvement [31,32,92,118,120,185]. However, these tiers require cautious interpretation because inflammatory and oxidative markers are affected by infection, acute illness, renal function, medication exposure, assay platform, and sample handling. Their value is therefore highest when interpreted as patterns of biological activity, not as isolated diagnostic thresholds.

Tiers 4 and 5 represent the most advanced and currently research-oriented levels of the framework. Tier 4 captures post-transcriptional regulation and intercellular communication, including inflammamiRs and extracellular-vesicle-associated miRNAs. These markers are conceptually important because they may indicate whether inflammatory and redox stress are being amplified, compensated, or poorly resolved. They also provide a bridge between local tissue stress and systemic signaling, especially in adipose tissue-immune cell-liver-muscle-endothelium crosstalk. Nevertheless, circulating and extracellular-vesicle miRNA assays remain limited by pre-analytical variability, hemolysis, normalization strategy, tissue-of-origin uncertainty and incomplete standardization [133,137,138]. Tier 5 includes metabolomic, lipidomic, proteomic, epigenetic and biological-aging signatures. These approaches may identify metabolic inflexibility, lipid toxicity, immune aging, organ vulnerability, and cumulative molecular damage before conventional disease endpoints emerge. Multi-omics studies are increasingly used to dissect insulin resistance, diabetes and cardiometabolic risk, but their clinical implementation remains constrained by cost, platform dependence, validation requirements, and the need for interpretable thresholds [31,185,186,187,188,189].

This framework should not be interpreted as a replacement for established MetS criteria or routine cardiometabolic care. Rather, it is a translational framework for organizing biomarkers according to clinical readiness and biological information. In practical terms, Tier 1 and selected Tier 2 markers are most suitable for near-term risk stratification and monitoring, whereas Tiers 3–5 are more appropriate for mechanistic phenotyping, longitudinal cohort studies, and predictive model development. The long-term goal is not to measure every marker in every patient, but to identify parsimonious biomarker combinations that classify patient phenotypes, improve monitoring, and predict progression toward T2DM, metabolic dysfunction-associated steatotic liver disease, cardiovascular disease, frailty, or multimorbidity. This staged approach is especially relevant in older adults, where chronological age, multimorbidity and polypharmacy can obscure the biological meaning of conventional metabolic markers. Biological-aging studies further support this logic by showing that molecular aging signatures may capture risk dimensions not fully represented by chronological age or routine clinical variables [190].

### 7.3. Candidate Biomarker Phenotypes

Clinical MetS is usually treated as a categorical diagnosis, but real patients present with substantial biological heterogeneity. This heterogeneity is already evident in the distinction between metabolically healthy obesity and metabolically unhealthy obesity: a systematic review and meta-analysis found that inflammatory markers, including CRP, IL-6 and TNF-α, differ across obesity phenotypes, indicating that adiposity alone does not determine inflammatory risk [191]. However, metabolically healthy obesity should not be interpreted as a permanent low-risk state; longitudinal studies indicate that it is often transient, with many individuals progressing toward metabolically unhealthy obesity or MetS over time [192]. Similarly, systemic inflammation varies across metabolic obesity phenotypes in population-based studies, supporting the view that clinically similar metabolic profiles can differ in inflammatory activity [193]. These observations align with the first two candidate phenotypes proposed here (Table 7): a metabolic-dominant compensated phenotype, in which conventional metabolic abnormalities occur with limited inflammatory-redox activation, and an inflammatory-metabolic phenotype, in which metabolic abnormalities are accompanied by active inflammatory tone.

The purpose of integrated biomarker phenotyping is not to diagnose MetS, but to predict clinically meaningful transitions. These include progression from compensated metabolic aging to T2DM, MASLD, cardiovascular disease, chronic kidney disease, sarcopenic obesity, frailty, multimorbidity and premature mortality. A second goal is to identify intervention-responsive biology, such as inflammatory-metabolic phenotypes that may respond strongly to weight loss, exercise, dietary quality improvement or anti-inflammatory strategies, and redox-injury phenotypes that may benefit from interventions restoring mitochondrial and endothelial resilience. Pharmacogenetic studies of metformin response in metabolic syndrome and T2DM further illustrate how biomarker-informed models may help stratify treatment response rather than simply describe baseline risk [196].

A second line of evidence comes from studies showing that MetS is not only metabolically heterogeneous, but also inflammatory–redox heterogeneous. Studies of inflammatory and oxidative status markers in MetS show that components are accompanied by variable patterns of inflammatory and oxidative biomarkers, rather than a uniform molecular profile [117]. This supports the proposed redox-injury phenotype, in which oxidative lipid, protein or DNA damage is prominent even when cytokine elevation is modest. Clinically, this phenotype is plausible because some patients present with vascular dysfunction, hepatic stress or tissue-damage risk that appears disproportionate to routine metabolic markers, suggesting that oxidative injury and impaired antioxidant reserve may define a distinct biological trajectory.

A third line of support comes from data-driven clustering. Recent cluster-analysis work in cardiometabolic disease identified distinct inflammatory phenotypes and even a discordant group with high cardiovascular burden but low TNF-α, suggesting that cardiometabolic risk cannot be reduced to a single inflammatory marker or one linear inflammation model [195]. Related cohort work has also used phenotype-based clusters to examine cardiometabolic complications and subclinical inflammation before T2DM diagnosis, reinforcing the idea that metabolic risk states can be classified by multidimensional biological profiles rather than by glucose or adiposity alone [197]. These findings support the logic of candidate phenotypes, but also warn that they should be tested empirically rather than assumed.

A fourth line of support comes from multi-omics studies. Multi-omics profiling in obesity and metabolic dysfunction has identified molecular heterogeneity involving inflammation, immunity, oxidative stress, lipid metabolism, metabolites, proteins, transcriptomic changes and miRNA-related regulation. For example, multi-omics work in childhood obesity identified distinct molecular clusters associated with obesity-related metabolic dysfunction, demonstrating that clinical obesity contains biologically separable subgroups [194]. Broader reviews of metabolic phenotyping also emphasize that metabolomics and related platforms can improve disease risk stratification and precision prediction across obesity, diabetes, and cardiovascular disease [198]. This evidence supports the inclusion of a regulatory-failure phenotype and an integrated high-risk inflammatory-redox phenotype, but these should be described as research-level constructs requiring validation.

On this basis, we propose five candidate biomarker phenotypes in age-associated metabolic dysfunction: the metabolic-dominant compensated phenotype, inflammatory-metabolic phenotype, redox-injury phenotype, regulatory-failure phenotype, and integrated high-risk inflammatory-redox phenotype. These phenotypes are not intended to replace established MetS definitions. Rather, they are hypothesis-generating constructs for organizing how conventional metabolic markers, inflammatory mediators, oxidative-stress biomarkers, and inflammamiRs may combine in real patients. Their clinical relevance should be tested against longitudinal outcomes such as incident T2DM, MASLD, cardiovascular events, frailty, chronic kidney disease, multimorbidity, and mortality.

The five candidate phenotypes proposed here should be regarded as author-derived, hypothesis-generating constructs. They were not generated from original longitudinal data, unsupervised clustering, latent-class modeling, or externally validated cohorts. Rather, they were developed by integrating evidence from heterogeneous mechanistic, translational, cohort, and biomarker studies. These phenotypes are therefore not intended to function as diagnostic categories, and their boundaries are likely to be overlapping rather than discrete. Their value lies in providing a conceptual structure for future empirical testing. Validation will require prospective cohorts with repeated biomarker measurements, standardized assays, clinically meaningful outcomes, and independent replication across populations differing in age, sex, adiposity, comorbidity burden, ethnicity, and medication exposure.

### 7.4. What Should Integrated Models Predict?

Age-associated metabolic dysfunction arises from nonlinear interactions among adiposity, glycemic burden, inflammation, oxidative stress, endothelial injury, and post-transcriptional regulation; therefore, conventional regression, although essential for hypothesis testing and effect estimation, may not fully capture correlated biomarkers, nonlinear thresholds, or higher-order interactions among biomarker tiers. Machine-learning approaches, including penalized regression, elastic net models, random forest, support vector machines, gradient boosting, clustering, and latent-class methods, can be used to test whether inflammatory, oxidative-stress, inflammamiR, or multi-omics markers improve risk stratification beyond routine clinical-metabolic variables. Because the five phenotypes proposed in this Review are theoretical, future studies should test whether comparable biological groups emerge empirically rather than assigning patients to these categories a priori.

Current evidence supports this direction but remains preliminary. In the China Suboptimal Health Cohort, metabolomic models based on 1011 plasma samples discriminated MetS with high performance, with reported AUC values of 0.887 for support vector machine, 0.993 for random forest, 0.914 for k-nearest neighbor, and 0.755 for logistic regression; implicated pathways included amino-acid, lipid and glutathione-related metabolism [199]. This finding supports the concept that higher-tier omics markers may capture metabolic-redox features not represented by routine clinical variables, although internal validation and case–control design limit direct clinical translation. Additional studies using inflammaging-related transcriptomic models in atherosclerosis, machine-learning prediction in older adults with early cardiovascular-kidney-MetS, and miRNA or molecular feature-selection approaches in T2DM and diabetic complications further support the relevance of inflammatory, vascular-aging, and post-transcriptional layers [200,201,202,203,204]. However, broader big-data reviews emphasize that many current models remain cross-sectional, internally validated, disease-specific, or computational, rather than externally validated tools for predicting progression of metabolic aging [205].

A practical validation strategy would be to collect longitudinal Tier 1–5 biomarker data together with clinical outcomes, then standardize variables, correct batch effects, address missing data and scale biomarkers before unsupervised analysis. Baseline biological profiles could be explored using k-means clustering, hierarchical clustering, Gaussian mixture models, consensus clustering, or latent profile analysis. For mixed categorical and continuous data, latent class analysis may be particularly useful. The number of clusters or classes should be selected using internal fit and stability metrics, such as silhouette width, gap statistic, Bayesian information criterion, entropy, bootstrapped stability, and biological interpretability. Longitudinal validation should then test whether these data-derived groups are stable, whether individuals transition between groups, and whether group membership predicts incident T2DM, MASLD progression, cardiovascular events, frailty, multimorbidity, or mortality. Latent transition analysis, group-based trajectory modeling and growth mixture modelling could be used to determine whether inflammatory, redox or inflammamiR-dominant trajectories emerge over time. Importantly, future studies should not force the data into five groups; rather, the proposed phenotypes should be treated as a reference framework and compared with empirically derived clusters [195,197,199,205].

Future studies should compare nested models—Tier 1 alone versus Tier 1 plus inflammatory, oxidative-stress, inflammamiR and multi-omics layers—and evaluate whether additional biomarkers improve discrimination, calibration, reclassification, and decision-curve performance. Explainability methods, including SHAP values, permutation importance, sparse penalized models and transparent risk scores, will be essential to ensure that predictions are driven by biologically plausible signals rather than dataset-specific noise. The most clinically useful outputs would be interpretable risk classes, biomarker-importance profiles and phenotype assignments that predict incident T2DM, metabolic dysfunction-associated steatotic liver disease, cardiovascular events, frailty, multimorbidity or mortality better than conventional metabolic markers alone. The proposed phenotypes would gain translational value only if similar biological patterns are reproducible across cohorts, improve prediction beyond routine clinical variables and remain interpretable after external validation.

## 8. Clinical Translation, Limitations and Validation Roadmap

Clinical translation of the Inflammaging–Redox–InflammamiR Axis should proceed as a staged validation problem rather than as immediate biomarker implementation. Conventional metabolic markers remain the clinical foundation, but inflammatory, oxidative-stress, endothelial, adipokine and inflammamiR layers may help identify which older adults with similar metabolic profiles are biologically unstable and more likely to progress toward T2DM, MASLD, cardiovascular disease, chronic kidney disease, frailty, sarcopenic obesity, multimorbidity, or premature mortality.

The clinical goal is therefore not to diagnose MetS, which can already be identified using routine criteria, but to predict progression, resilience, and intervention response. This distinction is central: integrated biomarkers should be judged by whether they improve risk prediction, calibration, reclassification, monitoring, or treatment selection beyond established clinical variables.

### 8.1. Translational Opportunities and Target Outcomes

A clinically useful biomarker framework should identify the dominant biology underlying risk, not merely classify individuals as high or low risk. The tiered model proposed here can be viewed as a translational scaffold: Tier 1 markers define the routine clinical-metabolic phenotype; Tier 2 markers add systemic inflammation, inflammaging and adipose endocrine-immune dysfunction; Tier 3 markers capture oxidative damage, antioxidant reserve, endothelial activation, prothrombotic biology and tissue remodeling; Tier 4 markers, including inflammamiRs and extracellular-vesicle-associated miRNAs, reflect post-transcriptional regulation and intercellular communication; and Tier 5 multi-omic and biological-aging signatures remain discovery-level tools for biological subtyping.

The goal of this framework is not to diagnose MetS, which can already be identified using routine clinical criteria. Its value lies in determining which older adults with similar metabolic profiles are biologically unstable and more likely to progress toward adverse outcomes. Relevant endpoints include transition from metabolically stable obesity to MetS, incident T2DM, MASLD progression, cardiovascular events, chronic kidney disease progression, frailty or sarcopenic obesity, cardiometabolic multimorbidity, all-cause mortality and response to lifestyle, metabolic or anti-inflammatory interventions. This is particularly relevant in settings where effective T2DM management remains a clinical and public-health challenge [206].

This distinction is essential because conventional markers define the visible metabolic phenotype, whereas inflammatory, oxidative-stress and inflammamiR-based markers may help identify the biological trajectory underlying that phenotype. For example, two older adults may have similar BMI, waist circumference, HbA1c and lipid profiles, but differ substantially in inflammatory tone, oxidative injury, endothelial stress, adipose dysfunction, and miRNA-mediated regulatory imbalance. Integrated biomarker profiling could therefore support risk stratification in older adults with borderline or intermediate metabolic risk, identify dominant disease axes such as inflammatory-metabolic, redox-dominant, endothelial-vascular, adipose-dysfunction, or regulatory-failure phenotypes, and monitor biological response to interventions such as weight loss, exercise, dietary change, glucose-lowering therapy or lipid-lowering treatment.

However, clinical adoption should require more than biological plausibility. A biomarker panel should improve prediction beyond established clinical variables, be reproducible across populations, show acceptable analytical performance and provide information that can influence clinical decisions. Accordingly, the Inflammaging–Redox–InflammamiR Axis should be validated against longitudinal and intervention-based endpoints rather than cross-sectional MetS status alone. Its translational value will depend on whether it improves prediction, risk reclassification, monitoring and clinical decision-making for diabetes, MASLD, cardiovascular disease, chronic kidney disease, frailty, multimorbidity, and mortality.

### 8.2. Limitations and Unresolved Questions

Several conceptual, biological, and methodological limitations constrain immediate clinical translation of integrated biomarker models. First, causality remains uncertain. Many inflammatory mediators, oxidative-stress products and circulating miRNAs are associated with obesity, insulin resistance, T2DM, vascular aging and multimorbidity, but association does not prove that they drive disease. Elevated IL-6, TNF-α, oxidized LDL, MDA, miR-155 or miR-21 may reflect pathogenic inflammatory-redox activation, compensatory stress responses, or downstream tissue injury.

Second, circulating biomarkers may not accurately reflect tissue biology. Plasma or serum IL-6, TNF-α, IL-1β, MDA, oxidized LDL, miR-155, miR-146a or miR-21 may not capture local processes in visceral adipose tissue, liver, skeletal muscle, pancreatic islets, or vascular endothelium. In older adults, this ambiguity is amplified by multimorbidity, renal and hepatic function, medications, and subclinical disease, all of which may influence biomarker release, distribution, and clearance.

Third, neither inflammation nor ROS should be interpreted as uniformly harmful. Persistent low-grade inflammation is linked to cardiometabolic risk, but inflammatory signaling is also required for host defense, tissue repair, immune surveillance, exercise adaptation, and metabolic remodeling. Similarly, ROS participate in mitochondrial adaptation, autophagy, vascular tone, innate immunity, and exercise-induced metabolic improvement. The clinical problem is therefore not inflammation or ROS per se, but the transition from adaptive signaling to persistent inflammatory-redox injury and loss of redox resilience.

Fourth, inflammamiRs are pleiotropic and context-dependent. A single miRNA may regulate multiple transcripts and exert different effects according to tissue, disease stage, metabolic state and extracellular-vesicle context. miR-146a may represent protective feedback or failed inflammatory resolution; miR-155 may promote macrophage-driven insulin resistance but also participate in host defense; and miR-21 may reflect repair, fibrosis, or chronic inflammatory remodeling. Therefore, inflammamiRs should not be labeled simply as “protective” or “pathogenic.” Their value is more likely to emerge from pathway-level interpretation and integration with cytokine, oxidative-stress, and clinical-metabolic markers.

A further limitation is that the literature contains important inconsistencies and context-dependent findings. Several inflammatory and oxidative-stress biomarkers show variable associations across populations, assay platforms, disease stages, and comorbidity profiles. Oxidative-stress biology is particularly complex: although oxidative damage is strongly linked to metabolic dysfunction, antioxidant interventions have not consistently translated into clinical benefit, underscoring the distinction between redox signaling, oxidative injury and therapeutic targetability. These inconsistencies reinforce the need to interpret biomarkers as context-dependent pathway signals rather than as unidirectional indicators of risk. They also highlight the unresolved question of causality because many biomarkers may reflect adaptive compensation or downstream tissue injury rather than primary drivers of metabolic aging.

Clinical translation is further limited by analytical, pre-analytical, and temporal variability. hs-CRP is standardized and inexpensive, but non-specific and affected by infection, smoking, obesity, autoimmune disease, and comorbidity [170,171]. Cytokines such as IL-6, TNF-α, and IL-1β are mechanistically informative but dynamic, low-abundance and sensitive to circadian rhythm, acute illness, sleep, stress, sampling conditions, medications, and assay platform [172,173,174,175]. Oxidative-stress markers also require caution: F2-isoprostanes are among the most reliable lipid-peroxidation markers, whereas MDA and TBARS are more accessible but less specific. Protein carbonyls, AOPPs, nitrotyrosine, 8-oxo-dG, GSH/GSSG, and antioxidant enzyme activities provide complementary information, but are influenced by sample matrix, storage, renal clearance, diet, smoking, antioxidant supplementation, inflammation, and assay specificity [118,119,120,121,122,176].

An additional unresolved issue is that the Inflammaging–Redox–InflammamiR Axis is likely to be modified by circadian–neuroendocrine regulation, which may influence both biological mechanisms and biomarker interpretation. Metabolic aging does not occur in a temporally neutral environment: glucose tolerance, insulin sensitivity, lipid handling, blood pressure, immune-cell trafficking, cytokine release, mitochondrial function, and oxidative-stress responses vary across the day, while circadian disruption has been increasingly linked to cardiometabolic and immune dysfunction [207,208]. Mechanistically, this interaction extends beyond sleep timing or behavior because core clock components operate within metabolically active tissues and immune cells. For example, macrophage Bmal1 regulates mitochondrial metabolism during inflammatory activation, linking circadian-clock machinery with immunometabolism, redox handling and inflammatory output [209]. Similarly, hepatic BMAL1 disruption modifies obesity-associated insulin sensitivity and liver disease, supporting a role for peripheral clocks in lipid and inflammatory-metabolic regulation [210]. Neuroendocrine signals add further complexity. Glucocorticoids are not simply pro-inflammatory or anti-inflammatory; they may suppress adipose-tissue macrophage inflammation while promoting adipocyte lipid accumulation and insulin resistance when exposure is chronic, excessive, or mistimed [211]. Conversely, melatonin acts as a darkness-linked mitochondrial-redox modulator, with reported effects on mitochondrial ROS, antioxidant signaling and mitochondrial dynamics, although its metabolic consequences depend on timing and context [212,213]. Human studies further show that circadian misalignment can impair skeletal-muscle insulin sensitivity and alter fatty-acid metabolism gene profiles, and that late eating during high endogenous melatonin exposure may worsen glucose tolerance, particularly in *MTNR1B* risk-allele carriers [214,215]. Therefore, future validation studies should standardize or model sampling time, sleep duration and quality, chronotype, shift-work exposure, light-at-night exposure, meal timing, physical-activity timing, glucocorticoid use and stress-related conditions when assessing inflammatory, oxidative-stress, adipokine and circulating miRNA signatures. Without such temporal and neuroendocrine context, part of the apparent heterogeneity in integrated biomarker profiles may reflect circadian phase or rhythm disruption rather than stable metabolic-aging biology.

Circulating and extracellular-vesicle miRNAs are particularly sensitive to methodological variation. Hemolysis, platelet contamination, blood-cell counts, anticoagulant choice, centrifugation, RNA extraction, extracellular-vesicle isolation, normalization strategy and PCR or sequencing platform can all influence measured abundance [163,177,178]. Studies of inflammamiRs should therefore report hemolysis control, sample processing, normalization strategy and whether miRNAs were measured in total circulation or defined extracellular-vesicle fractions. Total circulating miRNAs and EV-associated miRNAs should also be interpreted separately. Total plasma or serum miRNA measurements include mixed sources, such as protein-bound miRNAs, lipoprotein-associated miRNAs, platelet-derived signals, apoptotic bodies, and EV cargo, whereas EV-miRNA analysis attempts to capture vesicle-associated intercellular communication. These compartments may carry different biological meanings and should not be treated as interchangeable. For Tier 4B translation, a major bottleneck is EV isolation and characterization: ultracentrifugation, size-exclusion chromatography, immunoaffinity capture, and polymer-based precipitation can enrich different vesicle and non-vesicle fractions, resulting in variable miRNA profiles. Future studies should therefore report the isolation method, EV characterization markers, particle/protein ratio, hemolysis and platelet controls, normalization strategy and whether the analysis refers to total circulation, total EVs or defined EV subfractions [177,178].

Sex, menopausal status, and demographic heterogeneity represent additional modifiers that should be considered in future validation studies. In women, the menopausal transition is associated with estrogen decline, redistribution of adipose tissue toward greater central and visceral adiposity, adverse lipid and glucose changes, endothelial dysfunction and increased cardiovascular risk [216,217]. These changes may amplify adipose inflammation, oxidative stress, and vascular inflammatory signaling, thereby shifting some women from a metabolically compensated phenotype toward inflammatory-metabolic or redox-injury phenotypes. In men, greater lifelong visceral adiposity and differences in adipokine profiles, immune responses, and cardiometabolic risk trajectories may shape distinct inflammatory-redox patterns [218,219]. Therefore, sex, menopausal status, hormone therapy exposure, fat distribution, age-related changes in body composition and relevant demographic factors should not be treated only as adjustment variables. Instead, biomarker thresholds, phenotype assignment and predictive models should be tested using stratified or interaction-based analyses where sample size permits.

Finally, comorbidity and medication exposure are major sources of confounding. Chronic kidney disease, heart failure, liver disease, sleep disorders, cancer history, depression, frailty, sarcopenia, and polypharmacy can independently alter inflammatory tone, oxidative burden, miRNA release, and biomarker clearance. The biomarker phenotypes proposed in this review should therefore be regarded as hypothesis-generating constructs, not diagnostic entities. Future models must account for renal and hepatic function, medication exposure, frailty, physical activity, sleep, diet, comorbidity burden, sex, menopausal status, and temporal biology; otherwise, integrated biomarker frameworks risk remaining biologically elegant but clinically unhelpful.

### 8.3. Validation Roadmap for Integrated Biomarker Models

**Figure 5:**
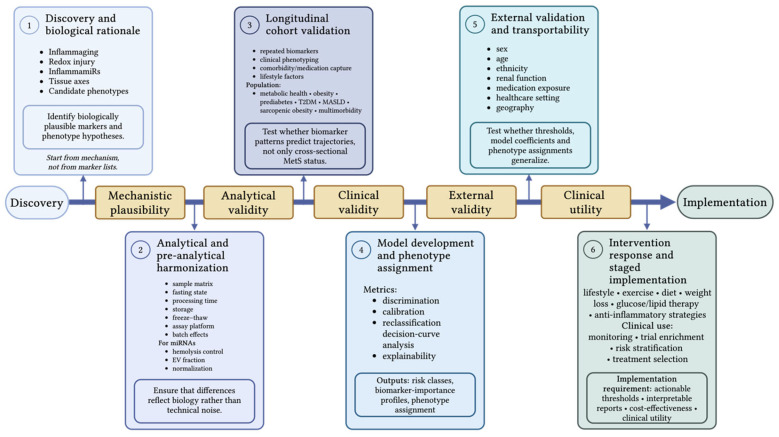
Translational roadmap for validating integrated metabolic-aging biomarkers. Clinical translation should proceed through staged validation: biological discovery, analytical standardization, prospective longitudinal cohorts, predictive modeling, external validation, and intervention-based implementation. Integrated models should compare Tier 1 clinical variables alone with added inflammatory, adipokine, oxidative-redox, endothelial, senescence-related, inflammamiR, and multi-omic layers. Candidate models should be judged by calibration, discrimination, reclassification, decision-curve performance, interpretability, and ability to predict clinically meaningful endpoints, including incident T2DM, MASLD progression, CVD events, CKD progression, frailty, multimorbidity, mortality and intervention response. Created in BioRender. Asanova, A. (2026) https://BioRender.com/rfi9z7t (accessed 21 May 2026).

The next priority should be validation rather than expansion of biomarker lists. Prospective longitudinal cohorts should include older adults across the spectrum of metabolic health, prediabetes, T2DM, obesity, sarcopenic obesity, MASLD and cardiometabolic multimorbidity. These studies should combine repeated biomarker measurements, standardized biospecimen collection, medication, and comorbidity assessment, and clinically meaningful outcomes such as incident diabetes, MASLD progression, cardiovascular events, chronic kidney disease, frailty, disability, multimorbidity and mortality. A staged translational roadmap for moving from biomarker discovery to clinical implementation is proposed in Figure 5. Although Figure 5 does not assign fixed time points to each stage, the roadmap can be interpreted as a set of approximate and overlapping translational phases. Discovery, biological rationale and analytical or pre-analytical harmonization are near-term priorities, because biomarker studies in multimorbidity, cardiovascular risk and miRNA profiling emphasize the need for standardized sampling, assay reproducibility, and clinically interpretable measurement before broader validation [177,178,180,182]. Longitudinal cohort validation and model development represent medium-term priorities, requiring repeated biomarker assessment, prospective clinical outcomes and comparison with routine clinical-metabolic variables [180,182,197,198,199]. External validation, transportability testing, and interventional implementation are longer-term steps, because current multi-omics and machine-learning models in metabolic and cardiometabolic disease often remain internally validated, disease-specific or insufficiently tested for incremental clinical utility across populations and health-care settings [181,182,183,199,200,201,202,203,204,205]. These phases should therefore be viewed as an evidence-informed sequence rather than a fixed timeline; the duration of each step will depend on assay maturity, cohort availability, regulatory requirements, and demonstration of clinical usefulness.

Validation should proceed in sequential stages. First, analytical harmonization is required, including standardization of fasting state, sampling time, sample matrix, processing, storage, freeze–thaw exposure, and assay platform. Cytokine studies must address detection limits and batch effects; oxidative-stress assays must minimize artefactual oxidation; and miRNA studies must control hemolysis, normalization, and extracellular-vesicle fractionation. Second, biological validation should test whether candidate phenotypes correspond to measurable tissue or organ dysfunction, including insulin sensitivity, visceral adiposity, hepatic fat, endothelial function, arterial stiffness, inflammatory cell profiles, mitochondrial function, frailty indices, and biological-aging measures. Imaging, tissue biopsies, immune phenotyping, and omics studies may be particularly informative; functional immune assays may further complement circulating biomarker panels by capturing immune-cell responsiveness rather than static inflammatory marker concentrations [220].

Third, predictive validation should compare nested models: clinical-metabolic variables alone versus models adding inflammatory markers, oxidative-stress markers, inflammamiRs, and multi-omics features. Related precision-medicine fields illustrate the value of predictive modeling for translating multidimensional clinical and molecular data into individualized therapeutic strategies [221]. Performance should be assessed using discrimination, calibration, reclassification, and decision-curve analysis. Fourth, external validation should test whether model performance is stable across sex, age, ethnicity, adiposity patterns, renal function, medication exposure, and health-care settings. Fifth, interventional validation should determine whether biomarker-defined phenotypes predict differential response to exercise, dietary change, weight loss, cardiometabolic therapy or anti-inflammatory strategies.

Clinical implementation should remain cautious and staged. At present, Tier 1 markers remain the only fully clinical layer of the framework. Tier 2 markers such as hs-CRP and selected adipokines may be feasible in research-oriented clinical settings, but most cytokines, oxidative-stress markers, and inflammamiRs are not ready for routine use. Tier 3 and Tier 4 markers require assay harmonization, interpretable thresholds, and outcome-based validation, whereas Tier 5 multi-omics and biological-aging signatures remain primarily research tools. Health-economic feasibility is another major constraint. Because metabolic aging is common, indiscriminate measurement of broad Tier 3–5 panels would be difficult to justify in routine care. Oxidative-stress assays, circulating or extracellular-vesicle miRNAs and multi-omic profiles require specialized processing, platform-specific analysis and bioinformatic interpretation, increasing cost, turnaround time and implementation complexity. Therefore, higher-tier testing should not be viewed as population-wide screening, but as a research, high-risk subgroup, clinical trial enrichment or reflex-testing strategy after Tier 1 and selected Tier 2 assessment. Future studies should evaluate not only predictive accuracy, but also cost-effectiveness, budget impact, workflow feasibility and whether testing changes clinical management or improves outcomes sufficiently to justify added cost [222,223].

The most immediate applications are therefore research cohorts, mechanistic studies, clinical trial enrichment, and predictive modeling. Integrated biomarker profiling may help identify biological subgroups, test whether inflammatory-redox-miRNA signatures add value beyond conventional risk factors, and design more precise intervention trials by selecting participants with active inflammatory, oxidative, endothelial, or regulatory dysfunction. Future validation studies should also consider populations exposed to accelerated biological stress, such as cancer survivors treated with chemotherapy or radiotherapy, in whom metabolic disruption, inflammation, oxidative injury, and biological-aging markers may be clinically relevant [224]. For eventual clinical use, interpretability will be essential: models should clarify which biomarker tiers drive risk, whether predictions are biologically plausible and how results should influence monitoring or intervention.

Ultimately, the goal is not to identify a single “best” biomarker of metabolic aging, but to develop validated, interpretable, and actionable multidomain signatures that integrate metabolic burden, inflammatory tone, redox injury, endothelial involvement, adipose dysfunction, post-transcriptional regulation and tissue-remodeling signals. Comparative transcriptomic evidence also supports the broader biological relevance of mitochondrial-redox adaptation, showing tissue-specific and cross-species conservation of nuclear-encoded mitochondrial metal and cofactor gene expression across buffalo and human tissues [225]. Recent pan-mammalian transcriptomic evidence identifying LGALS3/galectin-3 among conserved markers of aging, mortality, and chronic disease further supports its consideration as a candidate tissue-remodeling and inflammatory-risk biomarker, although it remains hypothesis-generating in the context of metabolic-aging stratification [226]. Until longitudinal and interventional validation is available, the Inflammaging–Redox–InflammamiR Axis should be presented as a conceptual and translational framework rather than a ready-to-use clinical algorithm.

## 9. Conclusions

Age-associated metabolic dysfunction is not only a disorder of glucose, lipids, adiposity, and blood pressure, but a biological state shaped by inflammaging, redox imbalance, adipose dysfunction, vascular aging, and post-transcriptional regulation. Substantial evidence supports the individual contributions of chronic low-grade inflammation, oxidative stress, mitochondrial dysfunction and miRNA-mediated regulation to metabolic disease and aging-related decline. The Inflammaging–Redox–InflammamiR Axis provides a framework for interpreting this heterogeneity: inflammatory mediators, oxidative-stress biomarkers and inflammamiRs are not isolated diagnostic candidates, but interconnected signals of immune activation, tissue injury, regulatory feedback, and metabolic adaptation.

The clinical value of this framework lies in predicting trajectory rather than naming disease. However, the organization of these mechanisms into a unified axis, as well as the proposed biomarker tiers and risk phenotypes, should be interpreted as author-derived conceptual hypotheses rather than validated clinical categories. Integrated biomarker models should identify which older adults with similar conventional metabolic profiles are most likely to progress toward T2DM, MASLD, cardiovascular disease, chronic kidney disease, frailty, multimorbidity or premature mortality—and which biological pathways may be most responsive to intervention.

Translation will require restraint. Inflammation is not always harmful, ROS are not merely damaging, and inflammamiRs are not unidirectional biomarkers. Each may reflect injury, adaptation, compensation or failed resolution depending on tissue context, timing, disease stage, and comorbidity burden. Longitudinal cohorts, external replication, and intervention studies are therefore essential before these models can guide clinical practice.

The next frontier is not to discover more isolated biomarkers, but to build interpretable and validated signatures of metabolic aging. If successful, this approach could transform metabolic risk assessment from a description of current abnormalities into a biological forecast of future vulnerability—identifying not only who is metabolically abnormal, but who is most likely to deteriorate, why, and how that trajectory might be modified.

## Figures and Tables

**Table 1:** Selected inflammatory biomarkers in age-associated metabolic dysfunction.

Biomarker Module	Representative Markers	Biological Axis Captured	Relevance to Metabolic Dysfunction	References
Systemic low-grade inflammation	CRP/hs-CRP; IL-6	Integrated inflammatory burden; IL-6-driven acute-phase response; SASP	Associated with obesity, visceral adiposity, insulin resistance, MetS, T2DM, frailty, cardiovascular risk, and inflammaging	[31,32]
Pro-inflammatory cytokine signaling	TNF-α; IL-1β	NF-κB/JNK activation, impaired insulin signaling, β-cell stress, and sterile inflammation	Linked to obesity-induced insulin resistance, adipose inflammation, impaired glucose uptake, T2DM, and MetS	[33,34,35]
Inflammasome activation	NLRP3 inflammasome activity; IL-18	Innate immune sensing, caspase-1 activation, and maturation of IL-1β/IL-18	Implicated in obesity, insulin resistance, β-cell dysfunction, MetS, T2DM risk, and cardiometabolic disease	[6,31,33]
Adipose immune-cell recruitment	CCL2/MCP-1	Recruitment of CCR2-positive monocytes/macrophages into adipose tissue	Reflects visceral adipose inflammation, macrophage infiltration, obesity, insulin resistance, and MetS	[34,36]
Adipokine imbalance	Adiponectin; leptin; resistin	Adipose endocrine dysfunction, altered insulin sensitivity, immune activation, and macrophage-associated inflammation	Low adiponectin, hyperleptinemia/leptin resistance, and increased resistin are associated with visceral obesity, insulin resistance, T2DM, fatty liver disease, MetS, and cardiovascular risk	[6,37,38,39,40]
Thrombo-inflammatory and fibrotic remodeling	PAI-1/*SERPINE1*; galectin-3	Prothrombotic inflammation, macrophage activation, fibrosis, endothelial dysfunction, and tissue remodeling	Associated with visceral obesity, insulin resistance, T2DM, cardiovascular disease, renal dysfunction, and age-related inflammatory remodeling	[41,42]

Note: This table summarizes selected representative inflammatory and immunometabolic biomarkers relevant to metabolic aging and is not intended to be comprehensive. These biomarkers should not be interpreted as isolated diagnostic indicators. Instead, they represent overlapping inflammatory axes linking cellular senescence, adipose tissue dysfunction, innate immune activation, insulin resistance, endothelial injury, and cardiometabolic remodeling during aging. Abbreviations: CCL2, C-C motif chemokine ligand 2; CCR2, C-C chemokine receptor type 2; CRP, C-reactive protein; hs-CRP, high-sensitivity C-reactive protein; IL, interleukin; JNK, c-Jun N-terminal kinase; MCP-1, monocyte chemoattractant protein-1; MetS, metabolic syndrome; NF-κB, nuclear factor-κB; NLRP3, nucleotide-binding oligomerization domain-like receptor family pyrin domain-containing 3; PAI-1, plasminogen activator inhibitor-1; SASP, senescence-associated secretory phenotype; SERPINE1, serpin family E member 1; T2DM, type 2 diabetes mellitus; TNF-α, tumor necrosis factor-α.

**Table 2:** Reframing metabolic syndrome as an inflammatory phenotype in older adults.

Classical Metabolic Feature	Conventional Clinical Interpretation	Inflammatory Reinterpretation	Key Biological Processes	Candidate Biomarker Signals	Clinical Implication	References
Central obesity/increased WC	Excess visceral adiposity and increased cardiometabolic risk	Visceral adipose tissue acts as an immune-metabolic organ producing chronic low-grade inflammation, especially in older adults with impaired adipose expandability and cellular senescence	Adipocyte hypertrophy; macrophage infiltration; adipose tissue hypoxia; SASP; NF-κB activation; inflammasome activation	↑ hs-CRP ↑ IL-6 ↑ TNF-α ↑ MCP-1/CCL2 ↑ leptin ↓ adiponectin ↑ leptin/adiponectin ratio	WC may indicate not only fat burden but also inflammatory adipose dysfunction; supports combining anthropometry with inflammatory/adipokine profiling	[36,37,38]
Elevated fasting glucose/impaired glucose tolerance	Pre-diabetes, insulin resistance, or T2DM risk	Glucose dysregulation reflects inflammatory interference with insulin signaling and β-cell stress driven by innate immune activation	JNK and NF-κB activation; macrophage-mediated cytokine release; IL-1β-mediated β-cell dysfunction; NLRP3 inflammasome activation; mitochondrial stress	↑ IL-6 ↑ TNF-α ↑ IL-1β ↑ IL-18 ↑ hs-CRP ↑ NLRP3-related signals ↓ adiponectin	Glucose elevation in older adults may represent an immunometabolic state; anti-inflammatory, weight-loss, exercise, and insulin-sensitizing interventions may target upstream biology	[6,33,35]
Hypertriglyceridemia	Atherogenic dyslipidemia, hepatic overproduction of VLDL, and increased cardiovascular risk	Triglyceride elevation reflects lipid overflow, adipose inflammation, hepatic inflammation, and immune activation by lipotoxic metabolites	Lipolysis from dysfunctional adipose tissue; ectopic lipid deposition; hepatic insulin resistance; inflammasome activation by lipids; macrophage foam-cell formation	↑ triglycerides ↑ ApoB/remnant cholesterol ↑ hs-CRP ↑ IL-6 ↑ TNF-α ↑ ferritin ↑ liver enzymes in fatty liver contexts	Hypertriglyceridemia can be interpreted as a marker of inflammatory lipid trafficking and ectopic fat stress, not simply excess circulating lipid	[6,33,38]
Low HDL cholesterol	Reduced reverse cholesterol transport and increased cardiovascular risk	Low HDL may reflect impaired anti-inflammatory, antioxidant, and cholesterol-efflux functions of HDL during chronic inflammation	HDL dysfunction; reduced cholesterol efflux capacity; oxidative modification of lipoproteins; macrophage activation; endothelial inflammation	↓ HDL-C impaired HDL function ↑ hs-CRP ↑ IL-6 ↑ oxidized LDL ↑ myeloperoxidase-related oxidative stress ↑ hs-CRP ↑ IL-6 ↑ TNF-α ↑ adhesion molecules ↑ VCAM-1/ICAM-1 ↑ galectin-3 ↑ PAI-1	In older adults, HDL quantity may be less informative than HDL function; low HDL may identify an inflammatory vascular-metabolic phenotype	[6,91]
Elevated blood pressure	Hypertension due to vascular stiffness, renal sodium handling, sympathetic tone, and endothelial dysfunction	Hypertension in aging can be viewed partly as vascular inflammation with immune-cell activation, endothelial dysfunction, and arterial stiffening	Endothelial activation; oxidative stress; vascular macrophage/T-cell infiltration; renin–angiotensin–aldosterone signaling; arterial stiffness; inflammaging	↑ hs-CRP ↑ IL-6 ↑ TNF-α ↑ adhesion molecules ↑ VCAM-1/ICAM-1 ↑ galectin-3 ↑ PAI-1	Blood pressure control may need integration with anti-inflammatory lifestyle strategies; inflammatory biomarkers may help identify older adults with vascular inflammatory risk	[31,32,92]
Insulin resistance	Reduced insulin sensitivity in muscle, liver, and adipose tissue; central driver of MetS	Insulin resistance is a tissue-level defense/adaptation to chronic nutrient excess and inflammatory stress, amplified by aging-related immune dysregulation	Serine phosphorylation of insulin receptor substrate proteins; macrophage cytokine signaling; mitochondrial dysfunction; ER stress; adipose tissue inflammation; impaired autophagy	↑ fasting insulin ↑ HOMA-IR ↑ IL-6 ↑ TNF-α ↑ hs-CRP ↑ MCP-1 ↓ adiponectin	Insulin resistance should be assessed alongside inflammatory status; exercise, weight loss, sleep improvement, and dietary quality may reduce both inflammation and insulin resistance	[33,34]
Non-alcoholic fatty liver disease/ectopic fat	Hepatic fat accumulation associated with insulin resistance and cardiometabolic risk	Fatty liver represents a hepatic inflammatory-metabolic phenotype involving lipotoxicity, innate immune activation, and systemic inflammatory signaling	Kupffer-cell activation; hepatocyte lipotoxicity; mitochondrial stress; inflammasome signaling; ER stress; altered gut-liver axis	↑ ALT/AST or GGT ↑ ferritin ↑ hs-CRP ↑ IL-6 ↑ TNF-α ↑ cytokeratin-18 in selected contexts	In older adults with MS, fatty liver may indicate systemic inflammatory lipid overflow and higher cardiometabolic vulnerability	[6,80]
Prothrombotic tendency	Increased cardiovascular and thromboembolic risk often associated with obesity, diabetes, and aging	Chronic metabolic inflammation promotes endothelial activation, impaired fibrinolysis, platelet activation, and immunothrombosis	Endothelial dysfunction; PAI-1 upregulation; platelet activation; complement activation; oxidative stress; monocyte activation	↑ PAI-1 ↑ fibrinogen ↑ D-dimer ↑ hs-CRP ↑ IL-6 ↑ platelet activation markers	Metabolic syndrome in older adults may be a pro-inflammatory and prothrombotic state; supports aggressive cardiovascular risk stratification	[31,41,92]
Sarcopenic obesity/reduced muscle quality	Coexistence of excess adiposity with low muscle mass or low muscle function; increased frailty and disability risk	Muscle–adipose crosstalk becomes inflammatory, with myosteatosis, cytokine signaling, mitochondrial dysfunction, and impaired anabolic response	Myokine imbalance; adipose infiltration into muscle; mitochondrial dysfunction; impaired autophagy; chronic IL-6/TNF-α exposure; anabolic resistance	↑ IL-6 ↑ TNF-α ↑ hs-CRP ↑ myostatin ↓ adiponectin altered irisin/myokine profile	In older adults, metabolic syndrome may overlap with frailty biology; interventions should prioritize resistance exercise, protein adequacy, and inflammation reduction	[31,32,37]
Clustering of MetS features	Diagnostic aggregation of abdominal obesity, dyslipidemia, hypertension, and impaired glucose regulation	MetS can be reframed as a systemic inflammatory phenotype driven by inflammaging, adipose immune remodeling, mitochondrial stress, and innate immune activation	Inflammaging; immunosenescence; senescent-cell secretory phenotype; adipose tissue inflammation; NLRP3 activation; endothelial dysfunction; oxidative stress	Multi-marker profile: ↑ hs-CRP ↑ IL-6 ↑ TNF-α ↑ IL-18 ↑ MCP-1 ↑ PAI-1 ↑ leptin ↓ adiponectin	Risk assessment may improve by adding inflammatory and adipokine markers to classical metabolic syndrome criteria, especially in older adults	[6,31,32]

Note: Arrows indicate expected direction of change in inflammatory or immunometabolic activation and should not be interpreted as diagnostic thresholds. Biomarker signals are intended to support biological interpretation, not replace established clinical criteria. Abbreviations: ALT, alanine aminotransferase; ApoB, apolipoprotein B; AST, aspartate aminotransferase; CCL2, C-C motif chemokine ligand 2; CKD, chronic kidney disease; CRP, C-reactive protein; ER, endoplasmic reticulum; GGT, gamma-glutamyl transferase; HDL, high-density lipoprotein; HDL-C, high-density lipoprotein cholesterol; HOMA-IR, homeostatic model assessment of insulin resistance; hs-CRP, high-sensitivity C-reactive protein; ICAM-1, intercellular adhesion molecule 1; IL, interleukin; JNK, c-Jun N-terminal kinase; LDL, low-density lipoprotein; MASLD, metabolic dysfunction-associated steatotic liver disease; MCP-1, monocyte chemoattractant protein-1; MetS, metabolic syndrome; NF-κB, nuclear factor-κB; NLRP3, nucleotide-binding oligomerization domain-like receptor family pyrin domain-containing 3; PAI-1, plasminogen activator inhibitor-1; T2DM, type 2 diabetes mellitus; TNF-α, tumor necrosis factor-α; VCAM-1, vascular cell adhesion molecule 1; VLDL, very-low-density lipoprotein; WC, waist circumference.

**Table 3:** Oxidative stress biomarkers and their relevance to metabolic dysfunction.

Oxidative-Stress Axis	Biomarkers	Biological Interpretation	Metabolic-Aging Relevance	Clinical Readiness	References
Lipid peroxidation and reactive aldehyde stress	F2-isoprostanes/8-iso-prostaglandin F2α; MDA/TBARS; 4-HNE	Reflects oxidative injury to polyunsaturated fatty acids and generation of reactive lipid-derived aldehydes with signaling and adduct-forming properties	Obesity, insulin resistance, dyslipidemia, T2DM, fatty liver disease, endothelial injury and vascular dysfunction	Moderate for research use; limited for routine clinical use. F2-isoprostanes are among the most robust systemic lipid-peroxidation markers, especially when measured by mass spectrometry, whereas MDA/TBARS are widely used but less specific. 4-HNE is mechanistically informative but analytically more demanding and less standardized for clinical application	[118,119,120,121]
Oxidative genomic stress	8-hydroxy-2′-deoxyguanosine/8-oxo-dG	Indicates ROS-mediated DNA oxidation and may capture oxidative pressure on genome maintenance and cellular senescence pathways	Aging, diabetes, obesity, vascular disease, cellular senescence, and chronic metabolic stress	Research-ready, but not clinically validated as a stand-alone marker. Urinary or circulating 8-oxo-dG is useful for assessing oxidative DNA damage in observational and mechanistic studies, but interpretation is influenced by DNA repair activity, renal clearance, assay platform and pre-analytical oxidation.	[119,120]
Oxidized lipoprotein injury	Oxidized LDL	Represents oxidative modification of LDL particles and pro-inflammatory, pro-atherogenic lipoprotein signaling	Dyslipidemia, insulin resistance, vascular inflammation, foam-cell formation, atherosclerosis and cardiometabolic risk	Translationally promising, especially for vascular-risk phenotyping, but not routine. oxLDL directly links oxidative stress with atherogenic vascular injury; however, assays are not fully standardized and levels are influenced by LDL burden, lipid-lowering therapy and vascular disease context	[6,118]
Protein oxidation, chlorination and nitration	Protein carbonyls; advanced oxidation protein products; nitrotyrosine/3-nitrotyrosine	Captures cumulative oxidative protein damage, myeloperoxidase-related chlorination and nitrosative stress affecting protein structure and function	Aging, mitochondrial dysfunction, inflammation, insulin resistance, frailty-related biology, renal dysfunction, endothelial dysfunction and vascular aging	Useful for mechanistic and cohort studies; low readiness for routine clinical decision-making. These markers capture complementary dimensions of protein oxidation and nitrosative stress, but they lack disease-specific thresholds and are affected by inflammation, renal function, sample handling and assay heterogeneity	[118,120,121,122]
Antioxidant reserve and redox buffering	GSH/GSSG ratio; total antioxidant capacity/total oxidant status; superoxide dismutase, catalase and glutathione peroxidase activity	Reflects redox-buffering capacity, systemic oxidant–antioxidant balance and functional antioxidant defense responses	Aging, obesity, diabetes, insulin resistance, fatty liver disease, mitochondrial dysfunction and inflammatory metabolic stress	Biologically informative but analytically context-dependent. These markers are useful for assessing redox resilience and antioxidant-system adaptation, but clinical interpretation is difficult because increased antioxidant activity may reflect compensation, whereas decreased activity may indicate exhaustion or deficiency. GSH/GSSG is particularly sensitive to sample handling.	[118,119,120,121]
Innate immune oxidative burst	Myeloperoxidase	Links neutrophil and monocyte activation to oxidant generation, LDL oxidation and endothelial injury	Vascular aging, atherosclerosis, endothelial dysfunction and cardiometabolic risk	Relatively advanced for vascular-inflammatory risk research, but non-specific. MPO links innate immune activation with oxidative vascular injury and has translational relevance in cardiometabolic and atherosclerotic contexts. However, it is strongly influenced by infection, acute inflammation, leukocyte activation and cardiovascular events, limiting specificity for metabolic aging.	[31,118]

Note: Oxidative-stress biomarkers are best interpreted as pathway-level indicators rather than disease-specific diagnostic markers. Lipid peroxidation products, oxidized lipoproteins, oxidatively modified proteins, DNA oxidation products and antioxidant-defense indices capture complementary aspects of redox imbalance in metabolic aging, but their clinical interpretation is strongly influenced by sample handling, assay specificity, renal function, diet, smoking, acute inflammation and comorbid cardiovascular or kidney disease. Abbreviations: 4-HNE, 4-hydroxynonenal; 8-iso-prostaglandin F2α, 8-iso-prostaglandin F2 alpha; 8-oxo-dG, 8-oxo-2′-deoxyguanosine; DNA, deoxyribonucleic acid; GSH, reduced glutathione; GSSG, oxidized glutathione; LDL, low-density lipoprotein; MDA, malondialdehyde; ROS, reactive oxygen species; T2DM, type 2 diabetes mellitus; TBARS, thiobarbituric acid-reactive substances.

**Table 4:** InflammamiRs relevant to metabolic aging.

Functional InflammamiR Axis	miRNAs	Biological Interpretation	Relevance to Metabolic Dysfunction	References
Inflammatory remodeling and tissue-stress adaptation	miR-21-5p	Stress-responsive miRNA linking macrophage, adipocyte and endothelial responses with PTEN–PI3K–*AKT*, TGF-β/SMAD, NF-κB, PDCD4 and AP-1 signaling	Associated with obesity-related cardiometabolic inflammation, adipose–immune crosstalk, diabetes-related tissue injury, redox-sensitive transcriptional responses and mitochondrial stress signaling	[133,134]
Innate immune negative-feedback control	miR-146a-5p	NF-κB-inducible feedback regulator that restrains TLR and IL-1 receptor signaling through IRAK1 and TRAF6 repression	Dysregulation may reflect insufficient anti-inflammatory compensation in diabetes, diabetic cardiomyopathy, impaired wound repair and adipose inflammatory states	[134,135,136]
Pro-inflammatory macrophage activation and insulin resistance	miR-155-5p	Immune-cell miRNA induced by inflammatory stimuli; promotes M1-like macrophage activation through NF-κB, JAK–STAT, SOCS1, PPARγ and TLR signaling	Macrophage-derived exosomal miR-155 may impair insulin sensitivity and reinforce adipose tissue inflammation, oxidative stress and cytokine amplification	[137]
Myeloid inflammasome regulation	miR-223-3p	Myeloid-enriched regulator of macrophage phenotype and NLRP3-related inflammatory signaling	Altered in visceral adipose tissue in obesity; implicated in adipose inflammation, inflammasome–ROS–cytokine crosstalk, insulin resistance and metabolic dysfunction	[138,139,140]
Diabetic extracellular-matrix and mitochondrial injury	miR-29a-3p/miR-29 family	Regulates ECM remodeling, insulin signaling, SIRT3-dependent mitochondrial function and high-glucose-induced tissue injury	Relevant to diabetic complications, impaired wound healing, mitochondrial dysfunction, oxidative injury and insulin-resistant tissue remodeling	[135,141,142]
Senescence-associated metabolic stress	miR-34a-5p	p53-linked, senescence-associated miRNA that suppresses SIRT1 and affects AMPK, PPARα, mitochondrial stress and apoptosis pathways	Implicated in NAFLD/MASLD, insulin resistance, β-cell stress, ROS accumulation, diabetes-related organ injury and age-associated metabolic decline	[143,144]
Endothelial repair and vascular aging	miR-126-3p/miR-126-5p	Endothelial-enriched miRNA supporting VEGF signaling, PI3K–*AKT* activity, vascular integrity and endothelial repair	Reduced miR-126 is associated with endothelial activation, oxidative vascular injury, impaired nitric oxide bioavailability, diabetic vascular disease and cardiometabolic risk	[145,146]
Oxidative vascular injury and diabetic tissue remodeling	miR-200 family, especially miR-200b/c	ROS-sensitive miRNA family linked to ZEB1/2, VEGF, SIRT1, Nrf2-related oxidative stress responses and TGF-β signaling	Relevant to diabetic vascular complications, endothelial dysfunction, wound healing, nephropathy, retinopathy biology and metabolic tissue injury	[145,147]
Inflammasome-sensitive cardiometabolic inflammatory signaling	miR-9-5p; miR-132-3p	Inflammation-responsive miRNAs connected to NF-κB, NLRP3-related signaling, cytokine networks, CREB, cholinergic anti-inflammatory pathways and insulin-sensitive tissue responses	May connect inflammatory signaling with oxidative and mitochondrial stress in diabetic cardiomyopathy, PCOS/metabolic inflammation and metformin-responsive metabolic-inflammatory states	[136,140]

Note: InflammamiRs should be interpreted as context-dependent regulatory signals rather than unidirectional markers of inflammation. Their biological effects depend on tissue source, cell type, disease stage, extracellular-vesicle carriage, metabolic state, and aging-related senescence burden. A given miRNA may indicate injury, adaptation, compensation, or failed resolution depending on the surrounding inflammatory, redox, and metabolic context. Abbreviations: *AKT*, protein kinase B; AMPK, AMP-activated protein kinase; AP-1, activator protein 1; CREB, cAMP response element-binding protein; ECM, extracellular matrix; IL, interleukin; IRAK1, interleukin-1 receptor-associated kinase 1; JAK–STAT, Janus kinase–signal transducer and activator of transcription; MASLD, metabolic dysfunction-associated steatotic liver disease; miR, microRNA; miRNA, microRNA; NAFLD, non-alcoholic fatty liver disease; NF-κB, nuclear factor-κB; NLRP3, nucleotide-binding oligomerization domain-like receptor family pyrin domain-containing 3; Nrf2, nuclear factor erythroid 2-related factor 2; PCOS, polycystic ovary syndrome; PDCD4, programmed cell death protein 4; PI3K, phosphoinositide 3-kinase; PPARα, peroxisome proliferator-activated receptor-α; PPARγ, peroxisome proliferator-activated receptor-γ; PTEN, phosphatase and tensin homolog; ROS, reactive oxygen species; SIRT1, sirtuin 1; SIRT3, sirtuin 3; SMAD, suppressor of mothers against decapentaplegic; SOCS1, suppressor of cytokine signaling 1; TGF-β, transforming growth factor-β; TLR, Toll-like receptor; TRAF6, TNF receptor-associated factor 6; VEGF, vascular endothelial growth factor; ZEB1/2, zinc finger E-box-binding homeobox 1/2.

**Table 5:** Cytokine-oxidative stress-inflammamiR feedback loops in age-associated metabolic dysfunction.

Feedback-Loop Component	Representative Mediators	Mechanistic Interpretation	Relevance to Aging	Relevance to Metabolic Dysfunction	Biomarker Implication	Reference
ROS-driven activation of inflammatory signaling	Mitochondrial ROS, NADPH oxidase-derived ROS, NF-κB, JNK, p38 MAPK	Excess ROS activate redox-sensitive inflammatory pathways, increasing transcription of cytokines and stress-response genes	Aging tissues accumulate mitochondrial dysfunction and weaker antioxidant buffering, making inflammatory activation easier to sustain	Promotes insulin-signaling impairment, adipose inflammation, endothelial dysfunction and hepatic lipid stress	Combined elevation of oxidative markers, such as F2-isoprostanes, MDA, 8-oxo-dG or protein carbonyls, with hs-CRP or IL-6 may indicate active redox-inflammatory stress	[31,118]
Cytokine-induced ROS production	TNF-α, IL-1β, IL-6, NOX enzymes, mitochondrial ROS	Pro-inflammatory cytokines increase ROS production through mitochondrial stress, NADPH oxidase activation and impaired antioxidant defenses	In older adults, chronic cytokine exposure may accelerate cellular senescence and tissue damage	Creates a feed-forward state in which inflammation worsens insulin resistance, β-cell stress, endothelial injury and adipose dysfunction	Concurrent increases in IL-6, TNF-α or IL-1β with MDA, oxLDL, 8-oxo-dG or nitrotyrosine may suggest cytokine-driven oxidative injury	[6,33]
Inflammasome-ROS amplification loop	NLRP3 inflammasome, caspase-1, IL-1β, IL-18, mitochondrial ROS	ROS and mitochondrial damage promote inflammasome activation; IL-1β and IL-18 then amplify inflammatory and metabolic stress	Aging is associated with mitochondrial damage, sterile danger signals and inflammasome priming	Contributes to β-cell dysfunction, insulin resistance, adipose inflammation and metabolic dysfunction-associated steatotic liver disease	IL-1β, IL-18, ASC/caspase-1-related signals plus oxidative stress markers may identify inflammasome-linked metabolic aging	[6,35]
NF-κB-miR-146a negative-feedback loop	NF-κB, miR-146a-5p, IRAK1, TRAF6, IL-1β/TLR signaling	NF-κB induces miR-146a, which suppresses IRAK1 and TRAF6 to restrain TLR and IL-1 receptor signaling	miR-146a is frequently discussed as an inflammaging-associated regulatory miRNA, reflecting attempts to contain chronic inflammatory tone	May limit excessive inflammation in diabetes and adipose inflammation, but persistent upregulation can indicate chronic immune activation	miR-146a together with IL-6, IL-1β, hs-CRP or oxidative markers may indicate compensatory anti-inflammatory feedback	[134,135]
Macrophage-derived miRNA-insulin resistance loop	Adipose tissue macrophages, exosomal miR-155, PPARγ, inflammatory cytokines	Inflammatory macrophages release miRNAs that alter insulin-sensitive tissues; miR-155 can impair insulin signaling partly through repression of metabolic regulatory pathways	Aging adipose tissue shows immune-cell remodeling and increased macrophage inflammatory tone	Links adipose inflammation directly to systemic insulin resistance	Circulating or extracellular-vesicle miR-155, together with TNF-α, IL-6 and HOMA-IR, may mark inflammatory insulin resistance	[137]
Adipose inflammation-miR-223 feedback loop	miR-223-3p, macrophages, NLRP3-related signaling, KLF6, cytokines	miR-223 modulates macrophage inflammatory phenotype and inflammasome-related activity; its upregulation in visceral adipose tissue may reflect counter-regulation of obesity-associated inflammation	Aging increases visceral adipose immune activation, making myeloid miRNAs relevant to inflammaging	Relevant to visceral adiposity, adipose macrophage activation, insulin resistance and MetS	miR-223 with MCP-1/CCL2, IL-6, IL-18 or NLRP3-related markers may indicate adipose immune activation	[138,139]
miR-21 inflammatory-remodeling loop	miR-21-5p, PTEN–AKT, PDCD4, TGF-β/SMAD, macrophage-derived extracellular vesicles	miR-21 is induced by inflammatory and oxidative stress and can regulate immune activation, fibrosis and tissue remodeling pathways	Aging tissues are prone to chronic repair responses, fibrosis and senescence-associated inflammatory signaling	May contribute to obesity-associated cardiometabolic inflammation, vascular dysfunction and diabetes-related tissue remodeling	miR-21 with hs-CRP, IL-6, PAI-1 or galectin-3 may indicate inflammatory-fibrotic metabolic remodeling	[133,134]
miR-34a-SIRT1/mitochondrial stress loop	miR-34a-5p, p53, SIRT1, AMPK, mitochondrial ROS	Metabolic and oxidative stress induce miR-34a; miR-34a can suppress SIRT1-related protective pathways, worsening mitochondrial dysfunction and inflammatory stress	miR-34a is linked to senescence-associated stress responses and impaired mitochondrial resilience	Relevant to hepatic steatosis, insulin resistance, β-cell stress and cardiometabolic complications	miR-34a with ALT/GGT, insulin resistance markers, oxidative stress markers or inflammatory cytokines may indicate hepatic/metabolic stress	[143,144]
miR-29a-mitochondrial inflammation loop	miR-29a, IL-6/STAT3, SIRT3, mitochondrial dysfunction	miR-29a participates in high-glucose inflammatory and mitochondrial stress responses; depending on tissue context, it may be protective or maladaptive	Aging impairs mitochondrial quality control and repair capacity, increasing vulnerability to miR-29-linked tissue dysfunction	Relevant to diabetic complications, wound healing, mitochondrial dysfunction and tissue repair failure	miR-29a with IL-6, mitochondrial stress markers or impaired wound/tissue-repair markers may indicate diabetic tissue aging	[135,141]
Endothelial oxidative inflammation-miR-126 loop	miR-126, VEGF signaling, PI3K-*AKT*, endothelial activation, VCAM-1-related pathways	Loss or dysregulation of endothelial miR-126 weakens vascular repair and promotes inflammatory endothelial dysfunction	Vascular aging involves endothelial dysfunction, oxidative stress and reduced repair capacity	Relevant to diabetic vascular disease, hypertension, impaired angiogenesis and cardiometabolic risk	Low miR-126 with oxLDL, nitrotyrosine, VCAM-1/ICAM-1 or endothelial microparticles may indicate vascular metabolic aging	[145,146]
SASP–redox-miRNA loop	Senescent adipocytes/endothelial cells, SASP cytokines, IL-6, TNF-α, miR-21, miR-34a, miR-146a	Senescent cells produce cytokines and ROS; these signals alter miRNA expression, which can further stabilize inflammatory, fibrotic and mitochondrial stress programmes	Central to inflammaging; senescence, ROS and cytokine production accumulate with age	Links metabolic dysfunction to chronic tissue remodeling, adipose dysfunction, vascular injury and frailty-related metabolic decline	A multi-marker panel combining SASP cytokines, oxidative damage markers and inflammamiRs may better capture active biological aging than a single biomarker	[31,32]
Extracellular-vesicle miRNA propagation loop	Exosomal miR-155, miR-21, miR-146a, miR-223; macrophage/adipocyte/endothelial EVs	Extracellular vesicles transfer miRNAs between immune and metabolic tissues, allowing local inflammation to become systemic	Aging is associated with altered intercellular communication and increased inflammatory signaling between tissues	Provides a mechanism by which adipose inflammation can alter liver, muscle, vascular and β-cell function	EV-associated miRNAs may be more informative than total circulating miRNAs for identifying tissue-specific inflammatory crosstalk	[133,137]

Note: The feedback loops shown are conceptual pathway models rather than validated diagnostic algorithms. Biomarker combinations should be interpreted as putative indicators of inflammatory-redox-miRNA network activity and require longitudinal, tissue-informed, and intervention-based validation before clinical use. Directionality may vary by tissue, disease stage, extracellular-vesicle compartment, metabolic context, and aging-related comorbidity burden. Abbreviations: 8-oxo-dG, 8-oxo-2′-deoxyguanosine; *AKT*, protein kinase B; ALT, alanine aminotransferase; AMPK, AMP-activated protein kinase; ASC, apoptosis-associated speck-like protein containing a CARD; CCL2, C-C motif chemokine ligand 2; CRP, C-reactive protein; EV, extracellular vesicle; GGT, gamma-glutamyl transferase; HOMA-IR, homeostatic model assessment of insulin resistance; hs-CRP, high-sensitivity C-reactive protein; ICAM-1, intercellular adhesion molecule 1; IL, interleukin; IRAK1, interleukin-1 receptor-associated kinase 1; JNK, c-Jun N-terminal kinase; KLF6, Krüppel-like factor 6; MAPK, mitogen-activated protein kinase; MCP-1, monocyte chemoattractant protein-1; MDA, malondialdehyde; miR, microRNA; miRNA, microRNA; NADPH, nicotinamide adenine dinucleotide phosphate; NF-κB, nuclear factor-κB; NLRP3, nucleotide-binding oligomerization domain-like receptor family pyrin domain-containing 3; NOX, NADPH oxidase; oxLDL, oxidized low-density lipoprotein; PAI-1, plasminogen activator inhibitor-1; PDCD4, programmed cell death protein 4; PI3K, phosphoinositide 3-kinase; PPARγ, peroxisome proliferator-activated receptor-γ; PTEN, phosphatase and tensin homolog; ROS, reactive oxygen species; SASP, senescence-associated secretory phenotype; SIRT1, sirtuin 1; SIRT3, sirtuin 3; SMAD, suppressor of mothers against decapentaplegic; STAT3, signal transducer and activator of transcription 3; TGF-β, transforming growth factor-β; TLR, Toll-like receptor; TNF-α, tumor necrosis factor-α; *TRAF6*, TNF receptor-associated factor 6; VCAM-1, vascular cell adhesion molecule 1; VEGF, vascular endothelial growth factor.

**Table 6:** Proposed tiered biomarker framework for age-associated metabolic dysfunction.

Tier	Biomarker Domain	Representative Markers	Biological Information Captured	Clinical Role	Key Limitation	References
Tier 1	Routine clinical-metabolic phenotype	Waist circumference, BMI, blood pressure, fasting glucose, HbA1c, fasting insulin/HOMA-IR, triglycerides, HDL-C, LDL-C, liver enzymes, eGFR	Captures conventional MetS, glycemic burden, dyslipidemia, adiposity, hepatic stress and renal function	First-line screening, diagnosis, risk stratification and longitudinal monitoring	Describes phenotype but does not identify inflammatory, oxidative or senescence-related mechanisms	[6,184]
Tier 2: Systemic inflammation and adipose dysfunction						
Tier 2A	Systemic inflammation and inflammaging	hs-CRP, IL-6, TNF-α, IL-1β, IL-18, MCP-1/CCL2	Captures chronic low-grade inflammation, innate immune activation, inflammasome activity and inflammatory tone	Helps distinguish metabolically abnormal but stable individuals from those with active inflammatory metabolic dysfunction	Cytokines are variable, non-specific and affected by infection, acute illness, medication and assay platform	[31,32]
Tier 2B	Adipose tissue endocrine and immune dysfunction	Adiponectin, leptin, leptin/adiponectin ratio, resistin, PAI-1, MCP-1/CCL2	Captures adipose expandability, adipocyte stress, adipose macrophage recruitment, insulin resistance and prothrombotic adipose dysfunction	Useful for interpreting central obesity as adipose inflammatory dysfunction rather than fat mass alone	Not routinely standardized in clinical practice; influenced by sex, fat distribution, renal function and medications	[37,38]
Tier 3: Oxidative, endothelial, and senescence-related injury						
Tier 3A	Oxidative stress and redox imbalance	F2-isoprostanes, MDA/TBARS, 4-HNE, 8-oxo-dG, protein carbonyls, nitrotyrosine, GSH/GSSG ratio, total antioxidant capacity, AOPP	Captures lipid peroxidation, oxidative DNA damage, protein oxidation, nitrosative stress and antioxidant reserve	Mechanistic phenotyping of the ROS-inflammation loop in metabolic aging	Many assays are pre-analytically sensitive; some markers lack specificity or clinical cut-offs	[118,120]
Tier 3B	Endothelial injury, vascular aging and prothrombotic signaling	VCAM-1, ICAM-1, E-selectin, oxidized LDL, myeloperoxidase, PAI-1, fibrinogen, D-dimer, galectin-3	Captures endothelial activation, oxidative vascular injury, impaired fibrinolysis, inflammatory-fibrotic remodeling and vascular aging	Useful in older adults with hypertension, diabetes, dyslipidemia, vascular disease or high cardiovascular risk	Not specific to metabolic dysfunction; affected by cardiovascular disease, infection, renal function and acute thrombosis	[92,185]
Tier 3C	Senescence-associated and tissue-remodeling signals	galectin-3, PAI-1, SASP-related IL-6/TNF-α, matrix-remodeling markers, selected fibrosis markers	Captures cellular senescence, inflammatory tissue remodeling, fibrosis, mitochondrial stress and reduced repair capacity	May help identify older adults in whom metabolic dysfunction overlaps with frailty, vascular aging, sarcopenic obesity or multimorbidity	Senescence markers are not tissue-specific and are not yet validated as routine clinical tools	[31,32,185]
Tier 4: Post-transcriptional regulation and intercellular communication						
Tier 4A	InflammamiRs	miR-21, miR-146a, miR-155, miR-223, miR-29a, miR-34a, miR-126, miR-200 family	Captures regulatory control of NF-κB, inflammasome signaling, macrophage activation, mitochondrial stress, endothelial dysfunction and senescence	Research-level inflammatory-redox phenotyping; may identify persistent cytokine-miRNA-oxidative stress feedback loops	Assay standardization, normalization, tissue origin and causal interpretation remain major challenges	[137,138]
Tier 4B	Extracellular-vesicle miRNA communication	EV-associated miR-155, miR-21, miR-146a, miR-223; adipocyte-, macrophage- and endothelial-derived EVs	Captures endocrine-like communication between adipose tissue, immune cells, liver, muscle and vascular endothelium	Helps conceptualize metabolic dysfunction as a systemic inter-organ communication disorder	EV isolation, purity assessment, quantification and cargo analysis are not standardized; different isolation methods may yield discordant miRNA profiles and limit routine clinical translation	[133,137]
Tier 5: Discovery-level multi-omics and biological-aging signatures						
Tier 5A	Metabolomic and lipidomic profiling	Branched-chain amino acids, acylcarnitines, ceramides, sphingolipids, phospholipids, bile acids, lactate/pyruvate-related metabolites	Captures mitochondrial substrate overload, lipid toxicity, insulin resistance, hepatic stress and altered nutrient flux	Precision phenotyping; may identify early metabolic inflexibility before overt clinical disease	Platform-dependent; requires validation, cost control and clinically interpretable thresholds	[186,187]
Tier 5B	Proteomic, epigenetic and biological-aging signatures	Inflammatory proteomic panels, DNA methylation age/pace-of-aging markers, organ-specific aging clocks, multi-omic aging scores	Captures integrated biological aging, immune aging, organ vulnerability and cumulative molecular damage	Research and future precision-medicine use; may stratify biological rather than chronological age in metabolic dysfunction	Not yet ready for routine MetS management; requires longitudinal validation and population-specific calibration	[31,185,188]

Note: The tiered framework is intended as a translational research model rather than a ready-to-use clinical algorithm. Tier 1 markers are established clinical measures, whereas Tiers 2–5 provide progressively deeper biological resolution but decreasing clinical validation. Selected Tier 2 markers may be clinically accessible but remain non-specific; Tiers 3–4 are mainly mechanistic or exploratory; and Tier 5 multi-omic and biological-aging signatures remain discovery-level tools. Tiers 3–5 are not proposed for routine population-wide screening and should be considered mainly for research cohorts, high-risk subgroup evaluation, clinical trial enrichment, or staged reflex testing. For Tier 4 markers, total circulating miRNAs and EV-associated miRNAs should be distinguished because they represent different biological compartments and are strongly affected by sample processing, EV isolation method, purity assessment, and normalization strategy. Higher-tier biomarkers require assay harmonization, longitudinal validation, external replication and demonstration of incremental predictive value beyond routine clinical variables before clinical implementation. Abbreviations: 4-HNE, 4-hydroxynonenal; 8-oxo-dG, 8-oxo-2′-deoxyguanosine; AOPP, advanced oxidation protein products; BMI, body mass index; CCL2, C-C motif chemokine ligand 2; CKD, chronic kidney disease; CRP, C-reactive protein; DNA, deoxyribonucleic acid; eGFR, estimated glomerular filtration rate; EV, extracellular vesicle; HbA1c, glycated hemoglobin; HDL-C, high-density lipoprotein cholesterol; HOMA-IR, homeostatic model assessment of insulin resistance; hs-CRP, high-sensitivity C-reactive protein; ICAM-1, intercellular adhesion molecule 1; IL, interleukin; LDL-C, low-density lipoprotein cholesterol; MASLD, metabolic dysfunction-associated steatotic liver disease; MCP-1, monocyte chemoattractant protein-1; MDA, malondialdehyde; miR, microRNA; miRNA, microRNA; NADPH, nicotinamide adenine dinucleotide phosphate; NF-κB, nuclear factor-κB; PAI-1, plasminogen activator inhibitor-1; ROS, reactive oxygen species; SASP, senescence-associated secretory phenotype; T2DM, type 2 diabetes mellitus; TBARS, thiobarbituric acid-reactive substances; TNF-α, tumor necrosis factor-α; VCAM-1, vascular cell adhesion molecule 1.

**Table 7:** Candidate metabolic aging phenotypes defined by integrated biomarker patterns.

Candidate Phenotype	Typical Biomarker Pattern	Biological Interpretation	Hypothesized Progression Risk	Relationship to Previous Literature	Clinical/Translational Implication
Metabolic-dominant compensated phenotype	Abnormal waist circumference, BMI, blood pressure, glucose, HbA1c, insulin resistance or lipids, but low inflammatory and oxidative-stress activity	Conventional metabolic burden without strong evidence of active inflammatory-redox injury	Low-to-moderate risk, but progression should be assessed longitudinally, especially if adiposity, insulin resistance or ectopic fat increases	Extends the concept of metabolically healthier obesity or lower-inflammatory metabolic states [191]	Standard metabolic monitoring may be sufficient initially, but progression should be assessed longitudinally
Inflammatory-metabolic phenotype	Metabolic abnormalities plus elevated hs-CRP, IL-6, TNF-α, IL-18, MCP-1/CCL2, PAI-1, galectin-3 or high inflammatory-cell indices	Metabolic dysfunction with active systemic or tissue-linked inflammatory tone	Higher risk of T2DM, MASLD, frailty and cardiometabolic multimorbidity, particularly when inflammation persists over time	Builds on evidence that inflammatory biomarkers differ across obesity and metabolic phenotypes [193]	Higher priority for cardiometabolic, frailty and multimorbidity monitoring
Redox-injury phenotype	Increased F2-isoprostanes, MDA, 4-HNE, oxLDL, protein carbonyls, AOPP, 8-oxo-dG or nitrotyrosine, with impaired antioxidant reserve	Oxidative tissue injury is prominent, even if cytokines are only moderately elevated	Higher vascular, hepatic and CKD progression risk, especially when oxidative markers coexist with endothelial injury or impaired renal function	Extends inflammatory–oxidative studies of MetS by separating redox injury from generic inflammation [117]	May identify vascular, hepatic, renal or tissue-damage risk requiring closer organ-specific assessment
Regulatory-failure phenotype	Increased miR-155 and miR-21, with reduced or insufficient compensatory miR-146a activity relative to inflammatory activation; context-specific changes in miR-223, miR-34a, miR-126 or miR-200 family members	Regulatory imbalance involving inflammatory amplification, failed resolution, endothelial injury or maladaptive tissue remodeling	Unstable trajectory, with potential progression toward inflammatory-redox amplification, diabetic complications or impaired intervention response	Extends multi-omics and miRNA biomarker studies by treating miRNAs as regulatory modifiers, not isolated diagnostic tests [194]	Promising for longitudinal prediction and treatment-response monitoring, but currently research-level
Integrated high-risk inflammatory-redox phenotype	Combined metabolic burden, inflammatory activation, oxidative damage, endothelial/prothrombotic markers, tissue-remodeling signals and inflammamiR dysregulation	Advanced inflammatory-redox-post-transcriptional metabolic dysregulation	Highest risk of cardiometabolic multimorbidity, frailty, CKD progression, cardiovascular events and premature mortality	Synthesizes inflammatory phenotyping, redox profiling, clustering and multi-omics into a single high-risk construct [195]	Highest priority for predictive modeling, closer monitoring and targeted prevention

Note: Candidate phenotypes are proposed as conceptual and translational constructs rather than validated diagnostic categories. They are entirely theoretical at present and were not derived from original clustering, latent-class modeling, longitudinal cohort analysis or externally validated datasets. Biomarker patterns should be interpreted longitudinally and in relation to clinical context, comorbidity burden, medication exposure, renal and hepatic function, frailty status and lifestyle factors. The same biomarker profile may reflect pathogenic activation, adaptive compensation or tissue injury depending on timing, tissue source, and disease stage. These phenotypes should therefore be regarded as a reference framework for future empirical testing, not as predefined clinical groups. These phenotypes require external validation before use in clinical decision-making. Abbreviations: 4-HNE, 4-hydroxynonenal; 8-oxo-dG, 8-oxo-2′-deoxyguanosine; AOPP, advanced oxidation protein products; BMI, body mass index; CCL2, C-C motif chemokine ligand 2; HbA1c, glycated hemoglobin; hs-CRP, high-sensitivity C-reactive protein; IL, interleukin; MCP-1, monocyte chemoattractant protein-1; MDA, malondialdehyde; MetS, metabolic syndrome; miR, microRNA; miRNA, microRNA; oxLDL, oxidized low-density lipoprotein; PAI-1, plasminogen activator inhibitor-1; TNF-α, tumor necrosis factor-α.

## Data Availability

No new data were created or analyzed in this study. Data sharing is not applicable to this article.

## Abbreviations

The following abbreviations are used in this manuscript:

4-HNE	4-hydroxynonenal
8-iso-prostaglandin F2α	8-iso-prostaglandin F2 alpha
8-oxo-dG	8-oxo-2′-deoxyguanosine
*AKT*	protein kinase B
ALT	alanine aminotransferase
AMPK	AMP-activated protein kinase
AOPP	advanced oxidation protein products
AP-1	activator protein 1
ApoB	apolipoprotein B
ASC	apoptosis-associated speck-like protein containing a CARD
AST	aspartate aminotransferase
BMI	body mass index
*CCR2*	C-C chemokine receptor type 2
CKD	chronic kidney disease
CREB	cAMP response element-binding protein
CRP	C-reactive protein
CVD	cardiovascular disease
DNA	deoxyribonucleic acid
ECM	extracellular matrix
eGFR	estimated glomerular filtration rate
ER	endoplasmic reticulum
EV	extracellular vesicle
*GDF15*	growth differentiation factor 15
GGT	gamma-glutamyl transferase
GSH	reduced glutathione
GSSG	oxidized glutathione
HbA1c	glycated hemoglobin
HDL	high-density lipoprotein
HDL-C	high-density lipoprotein cholesterol
HOMA-IR	homeostatic model assessment of insulin resistance
hs-CRP	high-sensitivity C-reactive protein
ICAM-1	intercellular adhesion molecule 1
IL	interleukin
IL-6	interleukin-6
inflammamiR	inflammation-related microRNA
IRAK1	interleukin-1 receptor-associated kinase 1
JAK–STAT	Janus kinase–signal transducer and activator of transcription
JNK	c-Jun N-terminal kinase
KLF6	Krüppel-like factor 6
LDL	low-density lipoprotein
LDL-C	low-density lipoprotein cholesterol
LPS	lipopolysaccharide
MAPK	mitogen-activated protein kinase
MASLD	metabolic dysfunction-associated steatotic liver disease
MCP-1	monocyte chemoattractant protein-1
MCP-1/CCL2	monocyte chemoattractant protein-1/C-C motif chemokine ligand 2
MDA	malondialdehyde
MetS	metabolic syndrome
miR	microRNA
miRNA	microRNA
MPO	myeloperoxidase
NADPH	nicotinamide adenine dinucleotide phosphate
NAFLD	non-alcoholic fatty liver disease
NF-κB	nuclear factor-κB
NLRP3	nucleotide-binding oligomerization domain-like receptor family pyrin domain-containing 3
NO	nitric oxide
NOX	NADPH oxidase
NOX4	NADPH oxidase 4
NRF2/Nrf2	nuclear factor erythroid 2-related factor 2
oxLDL	oxidized low-density lipoprotein
PAI-1	plasminogen activator inhibitor-1
PCOS	polycystic ovary syndrome
PCR	polymerase chain reaction
PDCD4	programmed cell death protein 4
PI3K	phosphoinositide 3-kinase
PPARα	peroxisome proliferator-activated receptor-α
PPARγ	peroxisome proliferator-activated receptor-γ
PTEN	phosphatase and tensin homolog
RNA	ribonucleic acid
ROS	reactive oxygen species
SASP	senescence-associated secretory phenotype
SERPINE1	serpin family E member 1
SIRT1	sirtuin 1
SIRT3	sirtuin 3
SMAD	suppressor of mothers against decapentaplegic
SOCS1	suppressor of cytokine signaling 1
STAT3	signal transducer and activator of transcription 3
T2DM	type 2 diabetes mellitus
TBARS	thiobarbituric acid-reactive substances
TGF-β	transforming growth factor-β
TLR	Toll-like receptor
TLR4	Toll-like receptor 4
TNF-α	tumor necrosis factor-α
TRAF6	TNF receptor-associated factor 6
VCAM-1	vascular cell adhesion molecule 1
VEGF	vascular endothelial growth factor
VLDL	very-low-density lipoprotein
WC	waist circumference
ZEB1/2	zinc finger E-box-binding homeobox 1/2
